# Synergy between silicon and beneficial microorganisms enhances plant abiotic stress tolerance through rhizosphere restructuring and coordinated defense

**DOI:** 10.3389/fpls.2026.1874835

**Published:** 2026-07-16

**Authors:** Jiaxin Cai, Yu Chu, Junli Wang, Pumo Cai, Yunjiang Liang, Yongcong Hong

**Affiliations:** 1 College of Agriculture, Yanbian University, Yanji, Jilin, China; 2 College of Tea and Food Science, Wuyi University, Wuyishan, Fujian, China

**Keywords:** abiotic stress, beneficial microorganisms, rhizosphere microorganisms, silicon, synergistic stress resistance

## Abstract

Abiotic stress is a major factor limiting crop productivity. Silicon (Si) and plant-beneficial microorganisms, as green agricultural inputs, exhibit significant limitations when applied individually. This review proposes the mechanisms by which their synergistic interactions enhance resistance to abiotic stress. Silicon optimizes the root microenvironment by regulating auxin-related genes, induces the formation of micropores in the root cortex, and promotes the secretion of photosynthates, thereby providing colonization sites and metabolic substrates for microorganisms. Beneficial microbes, in turn, activate mineral-bound silicon through the secretion of organic acids and upregulate host silicon-transport-related genes, coordinating the passive NIP aquaporin Lsi1 (gene encoding a NIP aquaporin-type silicon influx channel), which mediates monosilicic acid (H₄SiO₄) influx, with the active efflux transporter Lsi2 (silicon efflux transporter protein), thereby enhancing the uptake, loading, and internal distribution of bioavailable silicon in plants. At the physiological level, Si and beneficial microbes may cooperatively promote the physical co-deposition and spatial co-localization of silicon within lignin-enriched cell-wall regions, resulting in Si-associated lignified cell-wall reinforcement that strengthens apoplastic barriers and restricts harmful ion bypass flow. They may also jointly activate antioxidant networks, thereby alleviating stress-induced oxidative damage. Future research should integrate multi-omics approaches and machine learning to identify optimal “Si–microbe” combinations, and develop intelligent formulations based on porous nanosilicon carriers with environment-responsive release properties, thereby providing both a theoretical framework and technical pathway for the sustainable development of green agriculture.

## 1 Introduction

The constraints imposed by abiotic stress on crop growth, development, and yield formation are becoming increasingly severe, with stress regimes shifting from relatively single-stress conditions, such as drought, salinity, or heavy-metal toxicity, toward complex and combined stress scenarios (Cohen et al., 2021). This transition is primarily reflected in the joint effects of drought, extreme temperatures, and precipitation anomalies, which disrupt soil water–salt balance and accelerate the salinization and alkalization of arable soils (El-Ramady et al., 2024). Meanwhile, industrial emissions (Xu et al., 2021), wastewater irrigation (Othman et al., 2021), and mining activities (Xu et al., 2022) continue to drive the accumulation of heavy metals in agricultural soils, thereby further exacerbating risks to food security. Although conventional chemical-based stress-mitigation strategies can temporarily alleviate physiological damage caused by drought, salinity stress, or nutrient imbalance, their effectiveness is often influenced by soil pH, texture, and mineral composition. Moreover, high-input applications may increase environmental burdens and often fail to maintain stable performance under diverse field conditions (Mishra et al., 2023; Yuan et al., 2023; Zhang et al., 2025). Therefore, developing stress-mitigation strategies that are environmentally friendly, stable under field conditions, and mechanistically interpretable has become a critical priority in sustainable agriculture research.

Against this backdrop, the stress-mitigating potential of silicon and beneficial microorganisms has attracted sustained attention. Although silicon is not classified as an essential element for most higher plants, extensive evidence demonstrates that it enhances plant tolerance to stress by strengthening structural barriers, maintaining ionic homeostasis, and alleviating oxidative damage (Debona et al., 2017; Mehrabanjoubani et al., 2019; Meng et al., 2020). For instance, studies on wheat under drought stress have shown that exogenous silicon significantly alleviates oxidative and osmotic stress, improves leaf water status and antioxidant metabolism, and thereby enhances drought tolerance (ul Haq et al., 2024). Similarly, research on soybean under salt stress has demonstrated that silicon application maintains ionic balance and enhances antioxidant capacity, ultimately mitigating physiological damage caused by salinity (Peña Calzada et al., 2023).

Substantial evidence also supports the role of beneficial microorganisms in enhancing plant stress tolerance. Plant growth-promoting rhizobacteria (PGPR) and arbuscular mycorrhizal fungi (AMF) primarily improve plant adaptability to stress through hormonal regulation, nutrient mobilization, and modification of the rhizosphere environment (Munir et al., 2022; Bhupenchandra et al., 2025). For example, PGPR can reduce drought-induced ethylene accumulation via 1-aminocyclopropane-1-carboxylate (ACC) deaminase activity and promote root growth in grapevine (*Vitis vinifera* L.) through the production of indole-3-acetic acid (IAA) and other phytohormones (Duan et al., 2021). In addition, PGPR improve the rhizosphere microenvironment by secreting organic acids and extracellular polysaccharides (Ghosh et al., 2019), whereas AMF enhance the uptake of water and nutrients such as phosphorus through their extensive hyphal networks, thereby improving plant stress tolerance (Dere, 2024). Collectively, silicon and beneficial microorganisms enhance plant resilience through complementary mechanisms, including structural reinforcement, ion regulation, and rhizosphere-mediated biological modulation, forming a widely accepted conceptual framework.

However, the effectiveness of their individual application is often constrained by crop species, soil properties, and field ecological conditions, resulting in limited stability. Silicon fertilizers are susceptible to adsorption and fixation by clay minerals, iron and aluminum oxides, and fluctuations in soil pH, leading to reduced bioavailability in complex soil systems (Etesami, 2024). Meanwhile, exogenously introduced microbial strains often struggle to establish stable colonization under extreme stress conditions (Kong et al., 2025). To address these limitations, recent studies have explored the combined application of silicon and beneficial microorganisms for stress regulation in crops. For instance, Kamal et al (Kamal et al., 2025a). reported that the co-application of silicon and PGPR significantly improved drought tolerance in chickpea. Islam et al (Islam et al., 2026). demonstrated that the integrated use of silicon and AMF enhanced physiological and biochemical traits and increased yield in sunflower under salt stress. Additionally, under heavy metal stress, Khan et al (Khan et al., 2025). found that silicon synergistically alleviated metabolic damage and enhanced stress resistance in tomato grown in Cd-contaminated soils. Despite these advances, current mechanistic insights into silicon–microbe interactions largely remain at the level of observable effects—such as antioxidant enhancement, ion homeostasis, and growth recovery. Systematic evidence is still lacking regarding how silicon influences microbial recruitment and colonization, how microorganisms in turn regulate silicon bioavailability, and how their interactions are coordinated at the levels of transcriptomics, metabolomics, and rhizosphere signaling networks.

Unlike previous reviews that have separately focused on Si-mediated stress-tolerance mechanisms or the functions of plant growth-promoting microorganisms, this review emphasizes bidirectional feedback and cross-level synergistic mechanisms within the Si–beneficial microorganism–plant tripartite interaction. Specifically, the distinctive contributions of this review are threefold. First, it systematically discusses how Si improves microbial colonization niches and sustains microbial functional expression by regulating root architecture, the physicochemical properties of the rhizosphere, and root carbon allocation. Second, it summarizes how beneficial microorganisms enhance the bioavailability of mineral-bound Si and improve plant Si uptake efficiency through rhizosphere acidification, organic acid secretion, silicate solubilization, and regulation of plant Si transporter expression. Third, it proposes an integrated framework in which Si and beneficial microorganisms jointly regulate cell wall reinforcement, ionic homeostasis, antioxidant defense, and stress-related signaling pathways. On this basis, Si and beneficial microorganisms are not considered two independent categories of stress-mitigation inputs; rather, they are conceptualized as a composite regulatory system that connects rhizosphere Si cycling, the maintenance of microbial functions, and plant defense networks.

## 2 Mechanisms of silicon alone in mitigating abiotic stress

**Figure 1:**
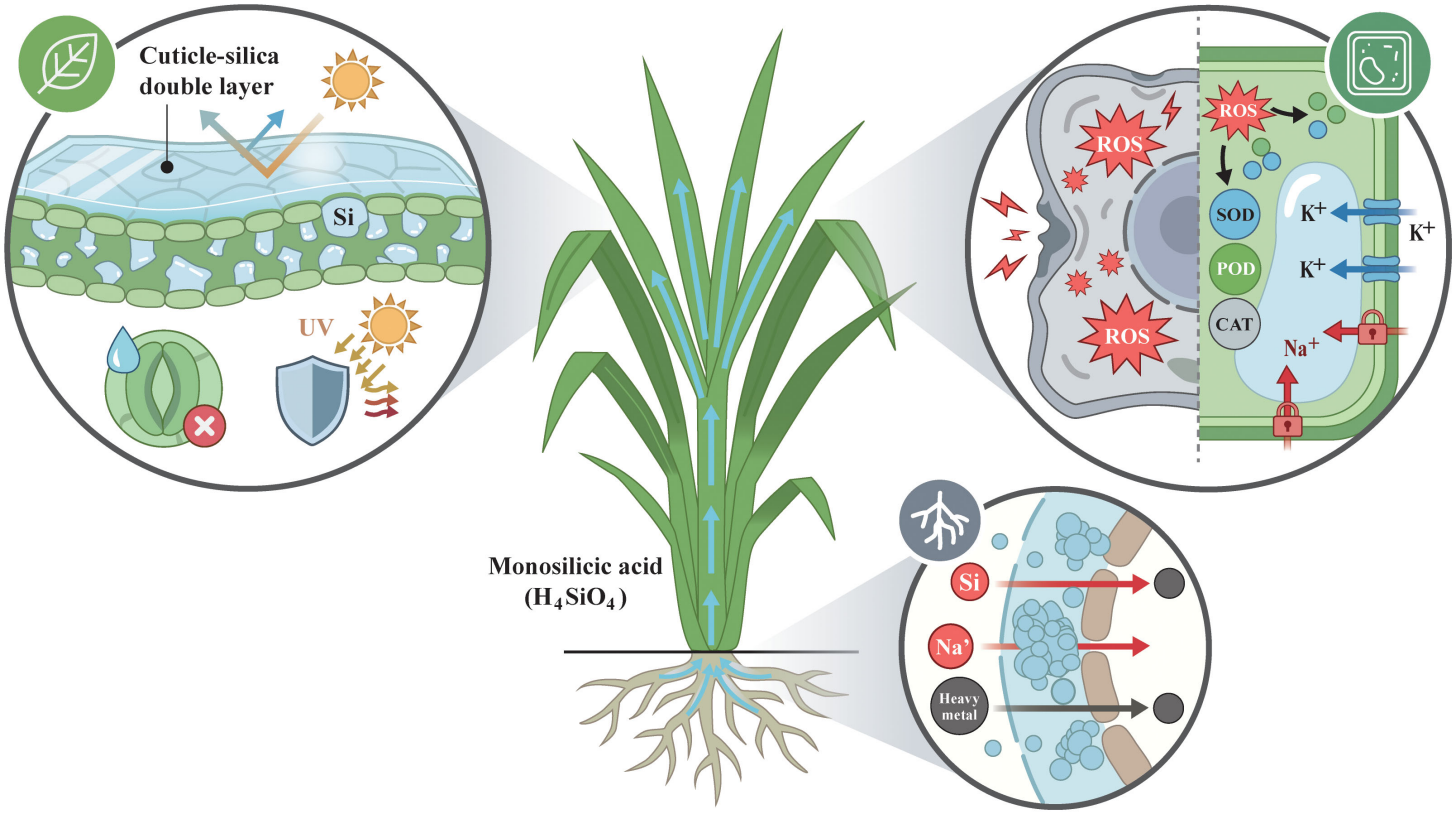
Conceptual representation of putative mechanisms by which silicon may contribute to plant tolerance to abiotic stress. The diagram summarizes major Si-associated processes reported in different plant systems, including structural reinforcement, ionic homeostasis, and antioxidant defense. Si may be deposited as amorphous hydrated silica in leaf epidermal tissues and root barrier regions, where it can contribute to reduced non-stomatal transpiration and restricted apoplastic bypass flow of toxic ions. Si may also modulate membrane transport, promote metal immobilization in cell walls or vacuolar compartments, and enhance enzymatic and non-enzymatic antioxidant networks involved in reactive oxygen species (ROS) scavenging. These mechanisms are not necessarily universal and may vary depending on plant species or genotype, Si accumulation capacity, Si source, dosage, developmental stage, and stress type. SOD, superoxide dismutase; ROS, reactive oxygen species; CAT, catalase; POD, peroxidase.

Silicon exerts multi-level regulatory effects in enhancing plant resistance to abiotic stress, encompassing the formation of structural barriers as well as the maintenance of ionic homeostasis and the regulation of antioxidant systems (**Figure 1**). These integrated mechanisms collectively constitute the fundamental basis by which silicon alleviates stress-induced damage.

### 2.1 Silicon deposition and the formation of structural barriers

The deposition of silicon in plant tissues is widely regarded as a key structural basis for its role in mitigating various abiotic stresses. Following uptake in the form of H₄SiO₄, silicon is deposited in the leaf epidermis and cell walls as amorphous hydrated silica, thereby enhancing tissue mechanical rigidity and reducing non-stomatal transpiration (Ma and Yamaji, 2006). In roots, silicon is primarily deposited in the exodermis and endodermis, often adjacent to the Casparian strip region, thereby reinforcing root barrier structures (Mandlik et al., 2020).

Overall, such deposition restricts the entry of toxic ions, such as Na^+^, into the stele via apoplastic bypass flow and reduces their translocation to the shoot, thereby alleviating salt stress in plants. For example, Yang et al (Yang et al., 2025a). and Fleck et al (Fleck et al., 2011), using rice silicon transporter mutants combined with tracer experiments, demonstrated that silicon application significantly reduced apoplastic bypass flow only in plants possessing a functional silicon transport system and effective root deposition. This suggests that the inhibitory effect of silicon on apoplastic bypass flow is not solely attributable to the structural barrier formed by silicon deposition, but likely depends on Lsi-mediated tissue-specific deposition and associated physiological regulatory processes.

Consequently, the research focus has shifted from whether Si forms a physical barrier to how it regulates the process of cell wall reinforcement. Substantial evidence suggests that Si modulates the expression of genes associated with lignin biosynthesis, including certain ABC transporters, thereby promoting the structural maturation of the Casparian strip (Zhao et al., 2021; Radotić et al., 2022; Rivai et al., 2022). This structural reinforcement represents a physical co-deposition of silicon in lignified cell walls, characterized by tight spatial co-localization within the matrix rather than the formation of novel chemical bonds. Furthermore, the structural reinforcement provided by Si deposition is not limited to root barriers. Schaller et al (Schaller et al., 2013). demonstrated that the silicified layer on the leaf surface not only reduces transpiration but also enhances the leaf’s shielding capacity against UV-B radiation by altering light scattering pathways, thus protecting the photosynthetic apparatus.

### 2.2 Physiological regulation and maintenance of ionic homeostasis

In addition to structural reinforcement, silicon contributes to the maintenance of ionic homeostasis through the regulation of membrane transport systems. Hurtado et al (Hurtado et al., 2021)demonstrated in sorghum (*Sorghum bicolor* L.) Moench) and sunflower (*Helianthus annuus* L.) that the combined application of root-supplied and foliar-applied silicon significantly improved Na^+^/K^+^ and Na^+^/Ca^2+^ ratios under salt stress, while maintaining higher K^+^ accumulation, thereby alleviating sodium toxicity.

Under heavy metal stress, the mitigating effects of silicon are typically associated with dual mechanisms involving cell wall immobilization and subcellular compartmentalization. On the one hand, silicon promotes the adsorption and co-precipitation of metals at cell wall binding sites, forming low-solubility complexes that reduce their bioavailability (Gu et al., 2012). On the other hand, silicon is often associated with increased metal sequestration in the cell wall and enhanced vacuolar compartmentalization, thereby decreasing their active concentrations in the cytoplasm and in sensitive tissues such as leaves, ultimately reducing systemic toxicity (Ulhassan et al., 2025). Further studies have shown that nanosilicon exhibits higher detoxification efficiency under Cd^2+^ stress in tomato (*Solanum lycopersicum* L.) (Irshad et al., 2026).

In addition, silicon can induce increased secretion of chelating organic acids, such as malate, from plant roots, thereby reducing the bioavailability of metals in the rhizosphere (Fan et al., 2016a).

### 2.3 Molecular regulation of the silicon transport system

The stress-mitigating functions of silicon largely depend on the spatial coordination of Lsi1 and Lsi2 across different root tissue layers (Ma et al., 2007). To be precise, Lsi1 functions as a passive nodulin 26-like intrinsic protein (NIP) aquaporin, facilitating the influx of H_4_SiO_4_, whereas Lsi2 operates as an active efflux transporter driven by a proton antiport system.In recent years, several studies have identified silicon transporters localized in the pericycle, revealing critical steps involved in the loading of silicon into the xylem for long-distance transport, and thereby providing new molecular evidence for silicon transport models (Gao and Chao, 2022; Huang et al., 2022; Yamaji et al., 2024a).

In contrast, the silicon transport system in dicotyledonous plants exhibits marked differences, underlining significant crop-specific variations in silicon accumulation. Studies in tomato (*Solanum lycopersicum* L.) have confirmed the presence of a functional Lsi1 influx channel in roots, but lack a fully functional Lsi2-mediated active efflux system. This molecular feature restricts the active loading of H_4_SiO_4_ into the xylem, explaining the relatively low silicon accumulation capacity observed in most dicot species compared to typical monocot silicon accumulators like rice (Sun et al., 2020a).

Notably, current molecular models are largely based on rice systems. For intermediate or low silicon-accumulating crops, it remains unclear whether non-canonical transport pathways or tissue-specific alternative mechanisms exist. Therefore, the regulation of Lsi1/Lsi2 expression should be understood as a Si-homeostasis process jointly shaped by shoot Si accumulation, phloem-mobile signals, local root nutritional status, and stress- or hormone-related signaling pathways (Mitani-Ueno et al., 2016; Huang and Ma, 2024; Yamaji et al., 2024b). However, there is still insufficient evidence for a single conserved Si-responsive cis-element or clearly identified transcription factor that directly controls Lsi1/Lsi2 expression. In legumes, the weakening or absence of the Lsi1/Lsi2 functional module should not be interpreted simply as complete gene loss, but may be associated with their low-Si-accumulation phenotype, the lack of an efficient Lsi2-mediated xylem-loading system, and an evolutionary strategy that relies more on nodulation-associated rhizosphere interactions than on Si deposition for stress adaptation (Sun et al., 2020b; Mitani-Ueno and Ma, 2021; Putra et al., 2022a). Addressing this question is essential not only for advancing the theoretical understanding of silicon nutrition physiology but also for determining the broader applicability of silicon in diverse crop systems.

However, the role of Si should not be understood solely in terms of structural barrier formation or antioxidant regulation within plants. Its effects on root architecture, cell wall properties, rhizosphere pH, and carbon source release may further alter microbial colonization sites, nutritional bases, and functional stability in the rhizosphere. This extended effect represents an important starting point for understanding the synergistic role of Si and beneficial microorganisms in enhancing plant tolerance to abiotic stress.

## 3 Independent mechanisms of beneficial microorganisms

**Figure 2:**
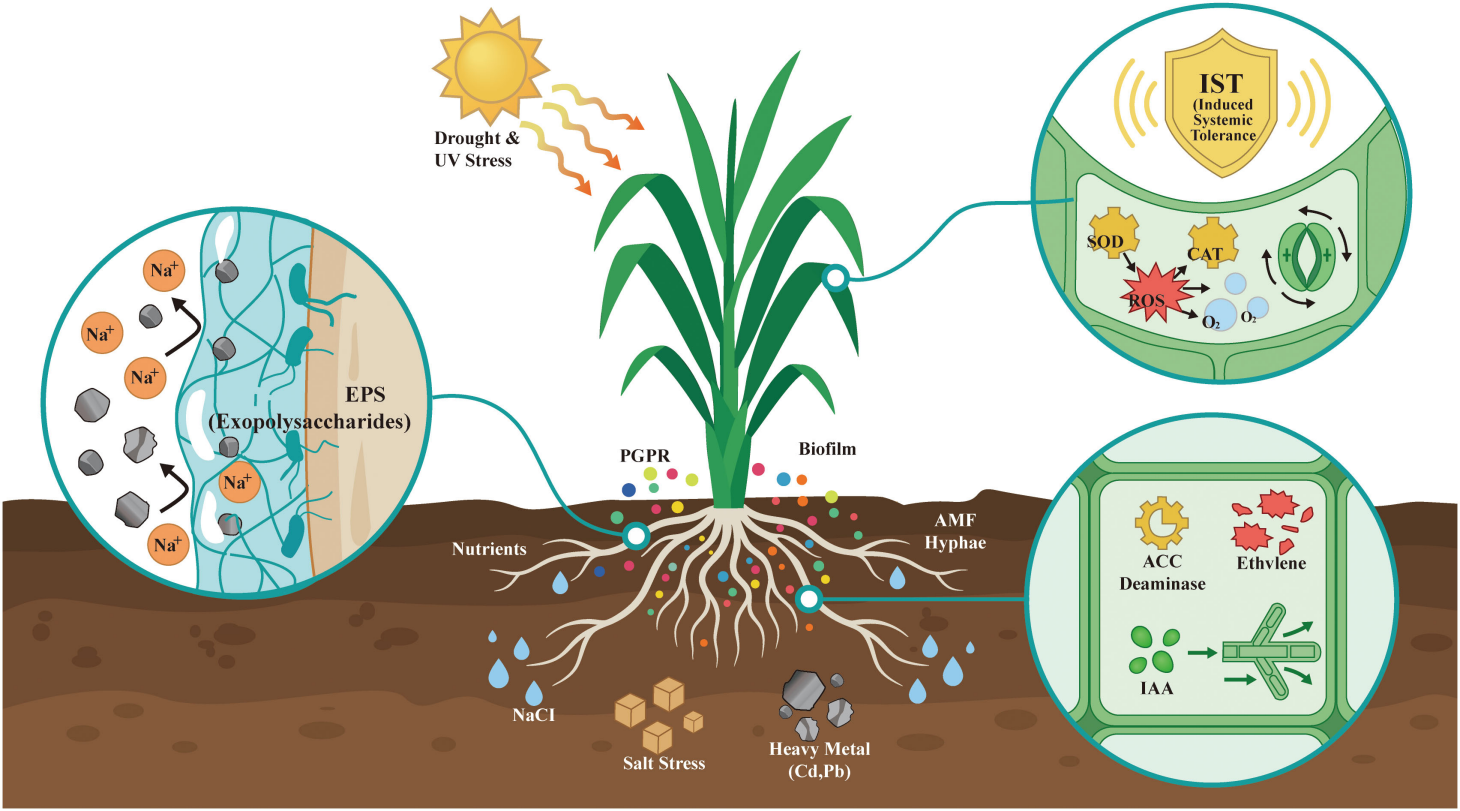
Conceptual representation of representative mechanisms by which beneficial microorganisms may enhance plant adaptation to abiotic stress. The diagram summarizes selected mechanisms reported for beneficial microorganisms, including hormonal modulation, rhizosphere chemical regulation, and root-surface protection. PGPR may reduce stress-induced ethylene accumulation through ACC deaminase activity and promote root growth through IAA production. Beneficial microorganisms may also mobilize mineral nutrients through organic acids, immobilize toxic ions through siderophores, EPS, or biofilm formation, and contribute to induced systemic tolerance (IST). These functions are strain-specific and condition-dependent, and their effectiveness may vary with microbial taxon, colonization ability, soil physicochemical properties, native microbial competition, host genotype, and stress intensity. EPS, extracellular polymeric substances; PGPR, plant growth-promoting rhizobacteria; AMF, arbuscular mycorrhizal fungi; SOD, superoxide dismutase; ROS, reactive oxygen species; CAT, catalase; ACC, 1-aminocyclopropane-1-carboxylic acid; IAA, indole-3-acetic acid; IST, induced systemic tolerance.

In contrast to the structural and physiological regulatory roles of silicon, beneficial microorganisms enhance plant stress tolerance primarily through hormonal regulation, rhizosphere modification, and activation of antioxidant systems (**Figure 2**), reflecting an adaptive strategy centered on biological regulation.

### 3.1 Hormonal regulation and root growth modulation

Beneficial microorganisms enhance plant adaptability to stress by modulating phytohormone balance, among which the 1-aminocyclopropane-1-carboxylate (ACC) deaminase pathway is one of the most representative mechanisms. This enzyme degrades ACC, the precursor of ethylene biosynthesis, thereby alleviating stress-induced overaccumulation of ethylene that leads to root growth inhibition and premature senescence (Bal et al., 2013; Barnawal et al., 2017).

Notably, the effects of this mechanism extend beyond hormonal regulation alone. For example, Peng et al (Peng et al., 2025). reported in licorice (*Glycyrrhiza uralensis Fisch*.) that the combined application of Bacillus and silicon significantly upregulated carbon metabolism-related pathways and reduced endogenous stress signaling under combined salt–drought stress, enabling plants to maintain relatively high root biomass even under extreme conditions. Furthermore, field trials using synthetic microbial communities (SynCom) by Wang et al (Wang et al., 2025). demonstrated that engineered microbial consortia can optimize rhizosphere microecological structure and enhance crop tolerance under stress conditions, providing important empirical support for translating laboratory findings into agricultural applications.

However, it should be noted that the hormone-regulatory effects observed under controlled laboratory conditions are often difficult to sustain in complex soil environments, primarily due to microbial colonization competition and environmental variability.

### 3.2 Rhizosphere chemical regulation and mineral nutrient mobilization

In stress-affected soils, one of the key functions of beneficial microorganisms is to enhance the bioavailability of mineral elements by reshaping the rhizosphere chemical environment. Various plant growth-promoting microorganisms can secrete low-molecular-weight organic acids (e.g., citric acid, oxalic acid), release H^+^, and produce phosphatases, thereby solubilizing insoluble phosphate complexes and certain iron oxides and converting them into plant-available forms (Sharma et al., 2013; Khanna et al., 2019).

These mechanisms have been substantiated at the field scale. In a maize field experiment, Wang et al (Wang et al., 2025)demonstrated using synthetic microbial communities (SynCom) that silicate-solubilizing bacteria not only promoted the conversion of mineral silicon into H₄SiO₄, but also significantly upregulated the expression of Lsi1 and Lsi2 in host roots. This finding indicates that microbial regulation of nutrient acquisition extends beyond soil chemical processes to the molecular regulation of host transport systems. Similarly, Etesami and Adl (Etesami and Adl, 2020) showed that phosphate-solubilizing bacteria (PSB) improved alfalfa growth under salt stress by alleviating the inhibitory effects of salinity on root nutrient uptake.

In recent years, research has increasingly focused on the ecological functions of siderophores. Microbially secreted high-affinity siderophores can chelate Fe^3+^ in the rhizosphere and facilitate its uptake by plants. Under heavy metal stress, these compounds can also alter the chemical speciation of metals through chelation, thereby reducing their toxicity (Wu et al., 2024; Zhang et al., 2026).

It is important to note that the organic acid-mediated solubilization effects observed under laboratory conditions are often constrained in field soils by soil buffering capacity. Soil pH, organic matter content, and mineral composition can neutralize or adsorb microbially secreted acidic substances, thereby diminishing nutrient mobilization efficiency (Jones, 1998; Keiluweit et al., 2015). Therefore, a key priority for future applications is the development of functional microbial consortia tailored to specific soil physicochemical conditions, enabling precise matching between the chemical environment and microbial functionality.

### 3.3 Extracellular polymeric substances and biofilm barriers

Beyond chemical transformations, beneficial microorganisms can influence plant responses to stress by modifying the root surface microenvironment and the physical structure of the rhizosphere. Many microbial strains are capable of secreting substantial amounts of EPS and forming biofilm structures on root surfaces, with EPS serving as the primary matrix (Santaella et al., 2008; Ge et al., 2022; Aoudi et al., 2024).

These biofilms not only enhance water retention in the rhizosphere but also reduce the bioavailability of Na^+^ and heavy metal ions at the root interface through electrostatic adsorption or complexation. For instance, Sun et al (Sun et al., 2020c), in their study of the endophytic bacterium Pantoea alhagi NX-11, reported that high EPS production was significantly associated with enhanced salt tolerance in rice seedlings, as reflected by reduced membrane lipid peroxidation and improved plant water status. Similarly, Xing et al (Xing et al., 2022). identified a heavy metal-tolerant plant growth-promoting rhizobacterium, Pseudomonas sp. H16, capable of forming stable biofilms. Functional groups such as carboxyl and hydroxyl groups within its EPS matrix can complex with heavy metal ions, thereby promoting their immobilization in the rhizosphere environment.

Unlike Si, which mainly contributes to structural barrier formation and the maintenance of physiological homeostasis within plants, beneficial microorganisms such as PGPR, AMF, and endophytes enhance plant adaptation to salinity, drought, heavy-metal stress, and other adverse conditions primarily by regulating hormonal balance, mobilizing mineral nutrients, improving the physicochemical environment of the rhizosphere, and forming extracellular polysaccharide- and biofilm-based barriers. A systematic understanding of these fundamental mechanisms provides a basis for further analyzing how Si-induced changes in the rhizosphere environment affect microbial colonization and functional expression, as well as how beneficial microorganisms reciprocally participate in Si mobilization and plant Si uptake.

## 4 Limitations of the independent application of silicon or beneficial microorganisms

Si and beneficial microorganisms can significantly enhance plant tolerance to abiotic stress under controlled conditions, their performance in field environments is often unstable. This discrepancy reflects multiple constraints arising from soil chemical processes, microbial community dynamics, and plant resource allocation, making it difficult for single-factor interventions to maintain consistent effectiveness under complex stress conditions.

### 4.1 Low utilization efficiency of silicon fertilizers

A key reason for the persistently low utilization efficiency of silicon fertilizers in field conditions is that a substantial portion of H₄SiO₄ is rapidly adsorbed or immobilized by soil minerals before reaching the root system. Field studies have shown that Fe/Al oxides in acidic soils can adsorb soluble H_4_SiO_4_, maintaining available silicon at relatively low concentrations (Epstein, 1994; Haynes and Zhou, 2020).

At the plant level, prolonged exposure to high silicon supply can induce the formation of a dense silica layer on root surfaces and suppress the expression of Lsi1 and Lsi2 via negative feedback, thereby reducing transmembrane silicon transport efficiency (Shanmugaiah et al., 2023). In addition, conventional solid silicon fertilizers generally exhibit low utilization efficiency within the application season, as a large fraction of silicon re-polymerizes and precipitates as amorphous silica before being absorbed by plants (Li et al., 2023).

These findings indicate that the limitations of silicon fertilizer efficacy arise not only from precipitation and adsorption processes at the soil interface but also from plant-mediated regulation of transmembrane transport, which together hinder the reproducibility of silicon supplementation effects observed under laboratory conditions in field settings (Tounkara et al., 2020). At present, there remains a lack of systematic quantitative research on how to maintain stable concentrations of bioavailable silicon in the rhizosphere. In particular, the phase transformation kinetics of silicon and the thresholds for plant uptake at the soil–root interface under different soil types and fertilization regimes require further elucidation.

### 4.2 Instability of microbial colonization

The stress-mitigating functions of exogenously applied plant growth-promoting microorganisms depend on their stable colonization; however, multiple field stressors—such as salinity, drought, and heavy metal contamination—often lead to rapid population decline. Studies have shown that under high salinity stress, the expression of biofilm-associated genes in plant growth-promoting Pseudomonas is significantly downregulated, thereby reducing its adhesion capacity on root surfaces (Akram et al., 2025). Under drought conditions, reduced soil moisture limits microbial mobility, consequently decreasing their efficiency in reaching and colonizing the rhizosphere (Backer et al., 2018).

Community-tracking studies further indicate that although exogenously applied microbial strains can initially achieve relatively high abundance, their relative abundance declines markedly within 2–3 weeks, making it difficult to maintain sufficient population density to sustain growth-promoting functions (Mosimann et al., 2017). These findings suggest that environmental filtering under extreme conditions, together with competition from indigenous microbial communities, jointly undermines the persistence of introduced strains, thereby limiting the stability of their beneficial effects in field environments.

At present, there is still a lack of systematic quantitative studies on colonization density thresholds and their variability under different stress regimes, as well as a shortage of ecological models capable of predicting the duration of functional persistence.

### 4.3 Resource trade-offs under combined abiotic stresses

In field environments, abiotic stresses typically occur in combination rather than isolation, meaning that a single regulatory pathway is often insufficient to meet the diverse physiological demands imposed by multiple stresses. As a result, plants must reallocate limited metabolic resources among key processes such as ionic homeostasis, osmotic adjustment, and antioxidant defense. Recent studies have shown that transcriptional and metabolic reprogramming induced by combined stresses is not merely an additive effect of individual stress responses, but instead involves specific network reconfiguration and resource trade-offs (Rivero et al., 2022).

For example, under combined salt–drought stress, silicon can reduce Na^+^ accumulation within plant tissues but has limited effects on drought-related physiological adaptation, resulting in noticeable constraints on plant growth (Ahmad et al., 2024). Similarly, in microbe-mediated pathways, the functional efficacy of PGPR is not consistently maintained under heavy metal stress. For instance, exposure to multiple heavy metals (Ni, Cd, Mn) significantly reduces ACC deaminase activity and indole-3-acetic acid (IAA) production in most microbial strains, with only a few retaining high activity under elevated metal concentrations (Carlos et al., 2016).

At the level of resource allocation, carbon and nitrogen utilization patterns under combined stress conditions differ markedly from those under single stresses, further indicating that strategies relying on a single regulatory pathway are unlikely to simultaneously optimize multiple physiological processes. Studies using recombinant lines of tomato (*Solanum lycopersicum* L.) have shown that improving nitrogen use efficiency can partially buffer the negative effects of multiple stresses on growth and yield, accompanied by coordinated reprogramming of nitrogen and carbon metabolic pathways (Lopez-Delacalle et al., 2020). Metabolomic and physiological analyses in tomato further revealed that under combined salt–high temperature stress, proline, ascorbate, and associated redox pathways are coordinately regulated, reflecting a reallocation of carbon fluxes to maintain redox balance and osmotic adjustment (Lopez-Delacalle et al., 2021).

## 5 Interactive mechanisms of silicon and beneficial microorganisms in alleviating abiotic stress

**Figure 3:**
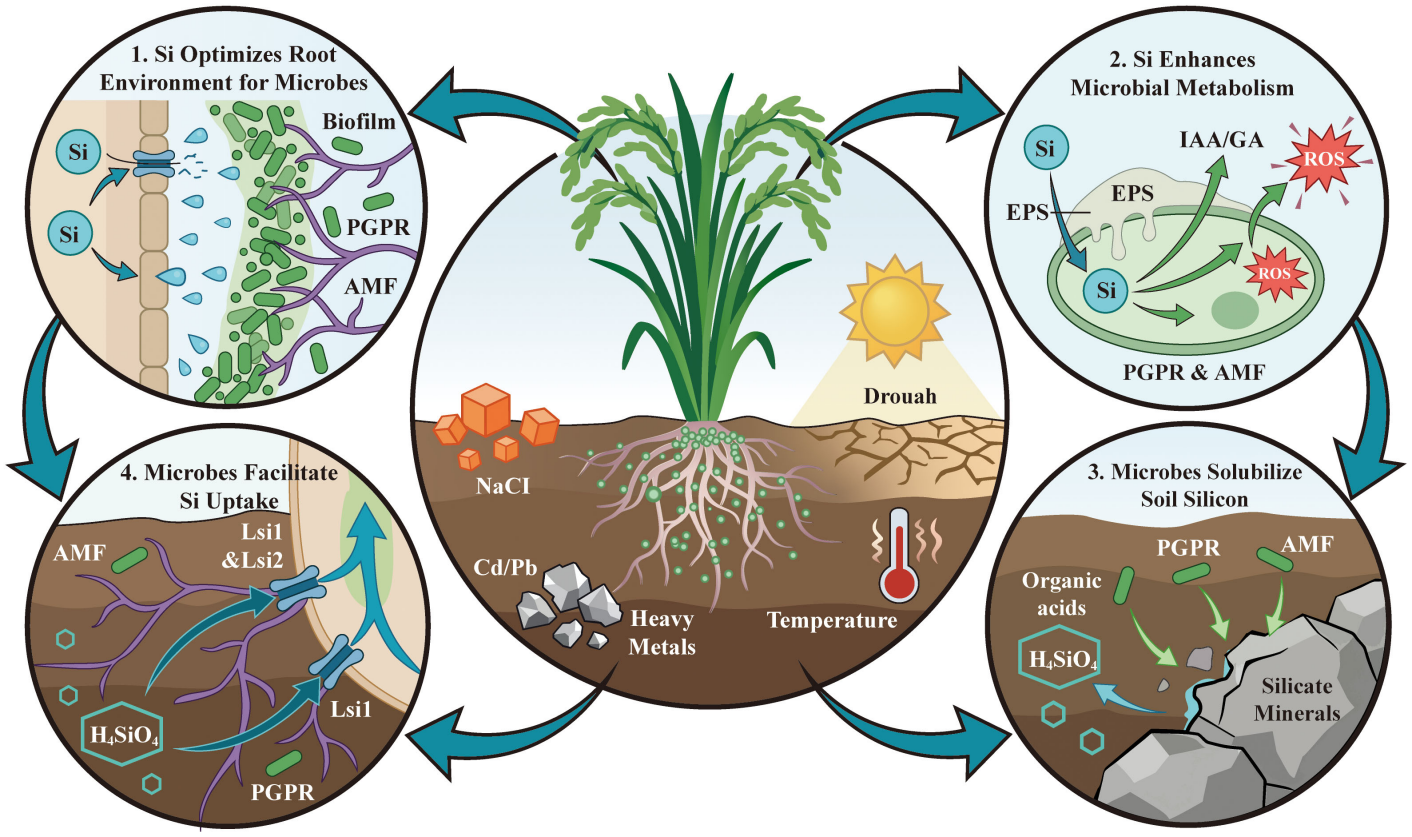
Proposed mechanisms underlying the synergistic alleviation of abiotic stress by silicon and beneficial microorganisms. The diagram presents a conceptual model of potential bidirectional feedback between Si, beneficial microorganisms, and plants. Si may facilitate microbial colonization and functional maintenance by modifying root architecture, root cortical traits, rhizosphere pH, and photosynthate-derived carbon supply. Conversely, specific microbial groups, such as silicate-solubilizing bacteria, PGPR, and AMF, may increase plant-available Si by promoting mineral weathering, organic acid secretion, and host Si transporter regulation. At the physiological level, combined Si–microorganism treatments may contribute to cell-wall reinforcement, ion homeostasis, and antioxidant defense under stress. The arrows represent proposed or evidence-supported associations rather than universally established causal pathways. The relative importance of each pathway may depend on crop Si accumulation capacity, Si source, microbial strain or consortium, soil background, and stress type and duration. PGPR, plant growth-promoting rhizobacteria; AMF, arbuscular mycorrhizal fungi; EPS, exopolysaccharides; ROS, reactive oxygen species; Lsi1/Lsi2, silicon transporter 1/2; IAA, indole-3-acetic acid; GA, gibberellic acid.

Si and beneficial microorganisms each have well-defined mechanisms for alleviating abiotic stress in plants; however, their individual application often suffers from insufficient stability under field conditions. To provide a clear overview of recent progress across different crop–stress–microbial taxon–Si source combinations, representative studies are summarized in **Table 1**, with distinctions made among stress type, crop species, Si source and dosage, microbial inoculant, experimental scale, major physiological effects, and Si-related evidence. This classification helps differentiate growth improvement, rhizosphere-mediated effects, and direct evidence of Si uptake under combined treatments. When Si and beneficial microorganisms are applied together, Si may influence microbial colonization and functional expression by altering plant root structure, the rhizosphere environment, and plant physiological status. Conversely, beneficial microorganisms may enhance Si uptake and utilization efficiency by mobilizing mineral-bound Si, improving rhizosphere conditions, and regulating plant transport and metabolic processes. Overall, this synergy involves the remodeling of the physical and chemical environment of the rhizosphere, regulation of nutrient cycling, maintenance of microbial functions, and coordination of plant signaling, metabolism, and physiological defense processes. However, this interaction should not be interpreted as a universal mechanism applicable to all crops, Si sources, microbial groups, and stress conditions. Instead, its strength and dominant pathways are largely determined by crop Si accumulation capacity, Si source characteristics, functional traits of the inoculant, soil background, and the type and duration of stress. Although the potential of Si and beneficial microorganisms to synergistically alleviate abiotic stress has been reported in diverse experimental systems, current evidence is still largely derived from short-term hydroponic, pot, or greenhouse studies. Therefore, the stability and reproducibility of this strategy at the field scale require further validation. Based on the available evidence, the major mechanisms underlying Si–microorganism interactions (**Figure 3**) can be discussed from the following perspectives:

**Table 1:** Summary of representative studies on the combined effects of silicon and beneficial microorganisms in mitigating abiotic stress.

Stress type	Drought stress	Salt stress	Salt stress	Salt stress	Salt stress	Salt stress	Drought stress	Salt stress	Salt stress	Sulfur-deficiency stress	Salt stress	Acidic/low plant-available Si soil context	Heavy metal stress (Ni)	Drought stress	Heavy metal stress (Cd/Zn)	Metalloid stress (As)	Salt stress	Drought stress	Metalloid stress (As)	Salt stress
Plant species	Brassica napus L.; Triticum aestivum L.	Coriandrum sativum L.	Zea mays L.	Phaseolus vulgaris L.	Vigna radiata L.	Vicia faba L.	Lens culinaris Medik.	Medicago sativa L.	Medicago sativa L.	Trifolium incarnatum L.	Cicer arietinum L.	Medicago sativa L. ‘Sequel’	Cicer arietinum; Vigna radiata; Cajanus cajan	Strawberry Fragaria × ananassa ‘Paros’	Cajanus cajan (L.) Millsp.	Cajanus cajan (L.) Millsp.	Citrullus lanatus L.	Glycyrrhiza glabra L.	Cajanus cajan (L.) Millsp.	Triticum aestivum L. ‘Pishiaz’
Silicon source and dose	Nano-Si, 100 and 200 mg·kg−1 soil; K2SiO3, 200 mg Si·kg−1 soil	K2SiO3, 0.1%	Exogenous Si, 3 mM	CaSiO3, 5, 10, and 15 g·kg−1 soil;RSM-optimized dose: 10.90 g·kg−1 soil	K2SiO3, 0, 1, and 2 kg Si ha−1	K2SiO3, 300 mg·L−1	Si, 0, 1, and 4 mM	K2SiO3, 0, 1, and 4 mM Si	CaSiO3, 3 mM Si	Na2SiO3, 1.7 mM Si	K2SiO3, 4 mM Si	Blast-furnace slag-based Si fertilizer, mainly Ca2SiO4; 0, 5, and 10 t·ha−1	K2SiO3, 300 mg Si·kg−1 soil	Na2SiO3, 3 mmol·L−1	K2SiO3, 300 mg·kg−1 soil	K2SiO4, 300 mg·kg−1 soil	Silicic acid, 2–4 mM;	SiO2, 300 mg·L−1	K2SiO3, 300 mg·kg−1 soil	Nano-Si, 0, 100, and 200 mg·kg−1 soil
Beneficial microorganism(s)	Pseudomonas sp. 19; Bacillus sp. 76; And their co-inoculation	Pseudomonas pseudoalcaligenes KB-10; Pseudomonas putida KB-25	Pseudomonas psychrotolerans CS51	Bacillus subtilis MTCC 441; Pseudomonas fluorescens MTCC 103	Enterobacter cloacae P6; Bacillus drentensis P16	Rhizobium leguminosarum bv. viciae TAL-1148; Bacillus circulans NCAIM B.02324	Rhizobium leguminosarum E10; Pseudomonas helmanticensis Rh23; Pseudomonas frederiksbergensis Rh32	Advenella incenata 40K; Ensifer meliloti N44	Ensifer meliloti Rm41	Rhizobium leguminosarum bv. trifolii T354	Funneliformis mosseae; Mesorhizobium ciceri PF:75	Ensifer meliloti RRI128	Rhizoglomus intraradices; Mesorhizobium ciceri PF:75; Rhizobium radiobacter VBCK1062; Sinorhizobium fredii AR-4	Rhizophagus clarus	Rhizophagus irregularis; Sinorhizobium fredii AR-4	Rhizophagus irregularis; Sinorhizobium fredii AR-4	Glomus mosseae; Gigaspora gigantea	Claroideoglomus etunicatum	Claroideoglomus etunicatum M1; Rhizoglomus intraradices M2	Bacillus subtilis SSB1; Pseudomonas fluorescens SSB2
Experimental scale	Greenhouse pot experiment	Soil-based pot experiment	Greenhouse pot experiment	Soil-based pot experiment	Field experiment	Field experiment	Greenhouse pot experiment	Greenhouse pot experiment	Growth-chamber substrate pot experiment	Hydroponic experiment	Soil-based pot experiment	Field experiment	Soil-based pot experiment	Growth-chamber substrate pot experiment	Soil-based pot experiment	Soil-based pot experiment	Greenhouse pot experiment	Greenhouse pot experiment	Greenhouse pot experiment	Soil-based pot experiment
Silicon-related evidence	Plant Si content was measured. In rapeseed, Si content was approximately 0.34% in the Si-free control, whereas 100 mg·kg−1 Nano-Si + Pseudomonas sp. 19 increased Si content to 4.05% under severe drought.In wheat, 200 mg·kg−1 Nano-Si + Pseudomonas sp. 19 increased Si content to 5.8% under severe drought.	No plant Si content, Si deposition, soil available Si, or Si transporter expression was measured.Si-related evidence was inferred from physiological responses to exogenous Si treatment; Therefore, the evidence is indirect.	Shoot Si content was measured. Under NaCl stress, Si + CS51 increased shoot Si to approximately 15 mg·g−1, compared with approximately 6–7 mg·g−1 in the NaCl control. Under non-saline conditions, Si + CS51 increased shoot Si to approximately 22 mg·g−1, compared with approximately 11–12 mg·g−1 in the control; Thus, this represents direct Si evidence.	No plant Si content, Si deposition, soil available Si, or Si transporter expression was measured.Si-related evidence was inferred from growth, yield, chlorophyll, and antioxidant responses to CaSiO3 treatment; Therefore, the evidence is indirect.	No plant Si content, Si deposition, soil available Si, or Si transporter expression was measured. Si-related evidence was based on the dose response of exogenous K2SiO3 and its interaction with PGPR; Therefore, the evidence is indirect.	No plant Si content, Si deposition, soil available Si, or Si transporter expression was measured.Si-related evidence was inferred from improvements in soil quality, ion homeostasis, and physiological responses after exogenous K2SiO3 treatment; Therefore, the evidence is indirect.	Soil Si concentration was measured. The S4 treatment significantly increased soil Si, reaching approximately 240 mg· kg−1 at 80–85% FC and approximately 170–180 mg·kg−1 at 50–55% FC. Plant Si content was not measured; Therefore, enhanced plant Si uptake should not be claimed.	Soil Si content was measured. Soil Si concentration was highest under 4 mM Si treatment, reaching approximately 14 mg·g−1; Under 15 dS·m−1 salt stress, it remained approximately 8–9 mg·g−1, higher than the Si-free treatment (approximately 4–5 mg·g−1). Plant Si content was not measured.	Plant Si content was measured. Application of 3 mM CaSiO3 increased alfalfa Si content from approximately 6–27 mg kg−1 DW to 3814–7450 mg kg−1 DW, providing direct Si evidence.	Root and shoot Si concentrations were measured. Under Si supply, root Si concentration was approximately 1.8–3.5 mg·g−1 DW and shoot Si concentration was approximately 1.1 mg·g−1 DW, providing direct Si evidence.	Root and leaf Si contents were measured. In HC3 under 100 mM NaCl, Si + AM increased root/leaf Si from approximately 0.5/1.1 to 1.1/2.3 mg·g−1 DW, providing direct Si evidence.	Soil available Si was measured. The 10 t·ha−1 treatment increased soil available Si by 181%. Leaf Si was measured by XRF but increased only marginally; Thus, the main direct evidence is soil Si availability.	Root and nodule Si contents were measured. Under Ni stress, +Si+AM increased nodule Si, reaching approximately 3.5 mg·g−1 DW in pigeonpea and 2.5 mg·g−1 DW in mung bean, providing direct Si evidence.	Leaf Si content was measured. The +Si+AMF treatment reached a maximum leaf Si content of 1.9 ± 0.1% DW and maintained 1.0 ± 0.1% DW under severe drought, higher than the Si-free control (0.1–0.5% DW), providing direct Si evidence.	Root and leaf Si contents were measured. The +Si+AM treatment increased leaf Si to approximately 5.5–5.6 mg·g−1 DW and root Si to approximately 2.2–2.3 mg·g−1 DW in pigeonpea, providing direct Si evidence.	Root and leaf Si contents were measured. Under As stress, +Si+AM still enhanced Si accumulation, increasing leaf Si from approximately 1.6–2.2 to 2.8–3.4 mg·g−1 DW, providing direct Si evidence.	No plant Si content, Si deposition, soil available Si, or Si transporter expression was measured. Si-related evidence was inferred from physiological responses to exogenous Si + AMF treatment; Therefore, the evidence is indirect.	No plant Si content, Si deposition, soil available Si, or Si transporter expression was measured. Si-related evidence was inferred from drought-responsive physiological changes after 300 mg·L−1 SiO2 treatment; Therefore, the evidence is indirect.	Leaf Si content was measured. The +Si+M2 treatment increased pigeonpea leaf Si under As stress to approximately 3.2–4.2 mg·g−1 DW, providing direct Si evidence.	Shoot and root Si contents were measured. Application of 200 mg·kg−1 Nano-Si increased shoot Si from 0.637% to 0.712% and root Si from 0.723% to 1.09%; Under SSB2 + earthworm treatment, shoot and root Si reached 0.744% and 0.924%, respectively.
Main physiological effects	① Increased antioxidant enzyme activities and reduced membrane lipid peroxidation; ② improved nutrient acquisition, plant growth, and water status; ③ Nano-Si combined with Pseudomonas sp. 19 showed a pronounced synergistic effect.	① Increased leaf RWC and maintained water balance; ② increased Chl a, Chl b, and carotenoids, protecting the photosynthetic apparatus; ③ enhanced POD activity; ④ reduced excessive polyphenol accumulation induced by salt stress; ⑤ increased biomass and salt tolerance index; ⑥ PGPB + Si outperformed single applications.	① Increased biomass and chlorophyll content; ② downregulated stress-related hormones, including ABA and JA; ③ increased flavonoids and total polyphenols while reducing proline accumulation; ④ reduced Na+ uptake and maintained ion homeostasis; ⑤ Si + CS51 was more effective than either treatment alone.	① PGPR + CaSiO3 increased plant height, pod length, and pod yield per plant; ② increased total chlorophyll content; ③ enhanced SOD and CAT activities; ④ the optimal combination was 5.52 × 107 CFU g−1 PGPR + 10.90 g·kg−1 soil CaSiO3.	① Increased stomatal conductance, transpiration rate, and RWC; ② increased photosynthetic pigment content; ③ reduced electrolyte leakage and enhanced membrane stability; ④ promoted plant height, leaf area, dry biomass, and seed yield; ⑤ 2 kg·ha−1 Si + B. drentensis P16 was the most effective treatment.	① Reduced soil ESP and increased urease and dehydrogenase activities; ② reduced leaf Na+ and increased K+, improving K+/Na+ homeostasis; ③ increased SPAD value, stomatal conductance, and RWC; ④ alleviated excessive antioxidant enzyme activation and osmotic stress; ⑤ increased pod number, 100-seed weight, seed yield, and N/P/K uptake.	① Increased plant height, root length, and shoot and root dry weight; ② increased nodule number and symbiotic efficiency; ③ increased N/P/K uptake; ④ improved leaf water status; ⑤ reduced proline and MDA accumulation, thereby alleviating osmotic stress and membrane lipid peroxidation.	① Salt stress reduced plant height, dry weight, root length, nodule number, chlorophyll, K, and N; ② increased shoot Na and CAT/SOD activities; ③ 40K and Si increased biomass, nodule number, chlorophyll, and RWC; ④ reduced Na while increasing K and N, thereby improving ion homeostasis and N nutrition.	① Increased biomass, chlorophyll, and RWC; ② increased nodule number, N content, and NCI; ③ reduced MDA, H2O2, and electrolyte leakage; ④ modulated SOD and PPO and increased polyphenols, flavonoids, carotenoids, and osmolytes; ⑤ reduced Na+ and increased K+.	① Increased root, stem, and whole-plant biomass; ② alleviated the inhibitory effect of sulfur deficiency on nodulation; ③ increased nodule number and nodule biomass; ④ increased nitrogenase abundance and N2 fixation; ⑤ promoted growth and symbiotic N fixation under sulfur deficiency.	① Salt stress increased O2−, H2O2, LOX, MDA, and ion leakage and disturbed the Ca2+/Na+ ratio; ② Si reduced stress metabolites and oxidative burst; ③ AM upregulated SOD, CAT, GPOX, and the AsA-GSH cycle; ④ Si + AM further reduced ROS accumulation.	① Increased soil available Si and pH; ② increased alfalfa yield by up to approximately 52%; ③ leaf Si showed only a weak increasing trend; ④ forage quality was not reduced; ⑤ altered arthropod community structure and increased functional diversity.	① Ni inhibited growth, N fixation, N assimilation, and yield; ② AM and Si both alleviated Ni toxicity; ③ AM improved soil enzyme activities and nutrient availability and reduced metal uptake; ④ enhanced nodulation potential, trehalose turnover, ammonia assimilation, and amide/ureide metabolism; ⑤ Si + AM was the most effective treatment.	① Increased biomass; ② increased photosynthetic rate, water content, and water-use efficiency; ③ enhanced antioxidant enzyme defense; ④ improved osmotic adjustment and micronutrient status, especially Zn; ⑤ Si + R. clarus alleviated drought-induced injury.	① Cd/Zn inhibited growth, chlorophyll, nutrient uptake, and yield; ② AMF improved root biomass and P/N/Mg/Fe status; ③ Si increased shoot biomass and K and Ca status; ④ AMF regulated proline metabolism; ⑤ Si + AMF improved water status, nutrition, and yield.	① As inhibited biomass, nutrient uptake, chlorophyll, and yield, with As(III) being more toxic; ② Si increased shoot biomass, whereas AMF increased root biomass; ③ AMF enhanced root and leaf Si uptake; ④ Si + AMF improved leaf water status, chlorophyll, biomass, and yield.	① Si + dual AMF increased leaf area, fruit size, fruit weight, harvest index, and carbohydrate content; ② increased chlorophyll, carotenoids, and K/Mg/P/Fe/Zn/Cu; ③ reduced osmotic potential, electrolyte leakage, and peroxide accumulation; ④ increased CAT, APX, SOD, and related antioxidant parameters.	① AMF and Si alleviated drought-induced growth inhibition; ② Si partially maintained plant performance at 20% and 40% FC; ③ improved plant height, membrane stability, and electrolyte leakage; ④ regulated MDA, SOD, POD, CAT, and other physiological and biochemical indicators.	① As inhibited AM symbiosis, nutrient uptake, growth, and yield, with As(III) being more toxic; ② Si and AMF increased soil glomalin and phosphatase activities and reduced As availability; ③ promoted nutrient acquisition, biomass, and chlorophyll accumulation; ④ enhanced starch-hydrolyzing enzyme activities and soluble sugars; ⑤ promoted proline biosynthesis.	① Salt stress reduced root length, shoot dry weight, LAI, RWC, chlorophyll, and K+ uptake, while increasing Na+, MDA, electrolyte leakage, and antioxidant enzyme activities; ② 200 mg·kg−1 Nano-Si increased root length by 44.87% and shoot dry weight by 21.52%; ③ reduced Na+, increased K+, and improved the Na+/K+ ratio; ④ SSB2 or SSB1 + earthworms promoted Si accumulation and salt tolerance.
Reference	(Valizadeh-rad et al., 2023)	(Al-Garni et al., 2019)	(Kubi et al., 2021)	(Kumar et al., 2020)	(Mahmood et al., 2016a)	(Hafez et al., 2021a)	(Kankia et al., 2025)	(Hosseini-Nasr et al., 2023)	(El Moukhtari et al., 2021)	(Coquerel et al., 2023)	(Garg and Bhandari, 2016)	(Putra et al., 2024)	(Thakur and Garg, 2023)	(Moradtalab et al., 2019)	(Garg and Singh, 2018)	(Garg and Kashyap, 2017)	(Bijalwan et al., 2021)	(Movahhed Haghighi and Saharkhiz, 2022)	(Bhalla and Garg, 2021)	(Fouladi et al., 2026)

The table organizes studies according to stress type (e.g., salinity, drought, and heavy metal exposure), plant species, silicon sources (including soluble forms and nanoparticles), and microbial inoculants (e.g., PGPR, AMF, and Rhizobium). Reported responses include changes in plant growth parameters, photosynthetic performance, ion homeostasis (e.g., Na^+^/K^+^ ratio), antioxidant enzyme activities, and nutrient uptake efficiency, as documented in the original studies. The attribution of observed effects to silicon–microbe synergy should be interpreted with caution, as many studies did not simultaneously measure plant silicon content, silicon deposition, transporter activity, or soil available silicon. Experimental systems vary substantially in scale (hydroponic, pot, greenhouse, and field conditions), species, and environmental settings.

### 5.1 Si-mediated regulation of the rhizosphere environment may promote microbial colonization and functional maintenance

Under salinity, drought, and heavy-metal stress, beneficial microorganisms such as PGPR and AMF often struggle to establish stable communities in the rhizosphere. Their colonization is constrained by multiple factors, including root structure, rhizosphere physicochemical conditions, and carbon source availability. Si application may alleviate these constraints through three interconnected dimensions: root structure, rhizosphere physicochemistry, and root exudation.

At the level of root structure, studies in specific plant systems have shown that Si is associated with changes in root traits. For example, in soybean, Si can increase root diameter, root hair density, and lateral root number by regulating the expression of genes involved in auxin biosynthesis and transport (Shamshiripour et al., 2022; Tripathi et al., 2022). These changes may provide additional attachment and colonization sites for certain symbiotic and rhizosphere bacteria. However, such responses should not be generalized to all crops. For species or genotypes with relatively low Si accumulation capacity, more evidence is needed to determine whether similar root structural responses occur and whether these responses are directly associated with Si deposition or indirectly related to improved water status, redox balance, or nutrient uptake.

The rice–AMF system provides a relatively clear example of how Si may influence microbial colonization. Studies have shown that inoculation with Rhizophagus irregularis combined with Si supply can increase AMF colonization and shoot Si accumulation in rice. Hyphal-compartment and isotope-tracing experiments further suggest that AMF hyphae may participate in Si uptake and transport in this system (Qiu et al., 2024a). However, rice is a typical high-Si-accumulating plant with a well-developed Si transport system; therefore, the same mechanism may not operate with comparable strength in low-Si-accumulating crops. For microbial groups other than PGPR and AMF, their relationships with Si-mediated rhizosphere regulation remain insufficiently investigated, although this issue is important for broadening the research scope of Si–microorganism interactions. In legume systems, nodulation and biological nitrogen fixation by Rhizobium and Bradyrhizobium depend on root-derived signals, nutrient supply, and a suitable redox environment. Si may indirectly support these processes by improving host water relations, antioxidant status, and nutrient balance; however, sufficient evidence is still lacking to demonstrate that Si universally enhances rhizobial colonization or nodulation.

Si may also affect microbial colonization by modifying root cell wall properties and cortical structure. Under specific stress conditions, particularly in high-Si-accumulating crops such as rice, Si deposition in lignified or suberized cell wall regions may strengthen root barriers and alter the cortical microenvironment. Such deposition not only enhances the mechanical strength of cell walls against osmotic stress but may also promote the formation of micropores in the root cortex by regulating programmed cell death (PCD) (Ghorbanpour et al., 2020; Soukup et al., 2020; Yan et al., 2021). In barley, Si nanoparticles have been reported to regulate root pore formation, with different dosages inducing distinct pore patterns, which may influence PGPR attachment or AMF hyphal expansion (Ghorbanpour et al., 2020). These phenomena are species- and dose-dependent, and root pore formation is also affected by multiple factors, including plant genotype, developmental stage, oxygen supply, stress intensity, Si formulation, and nanoparticle behavior on the root surface. Therefore, Si-mediated changes in cortical structure may promote microbial colonization in certain crop–microbe systems, but the magnitude and functional significance of this effect may vary across crop species and experimental conditions.

With respect to the rhizosphere chemical environment, Si can modify root exudates and rhizosphere pH, thereby affecting the chemotaxis and attachment of beneficial microorganisms. Under salt stress, Si treatment significantly increased soluble sugar content in cucumber roots and improved leaf photosynthesis and water status, which may have enhanced the availability of carbon-containing compounds in the rhizosphere and provided a more stable energy basis for rhizosphere bacteria (Zhu et al., 2016). However, only a limited number of systems have directly demonstrated a link among Si-induced photosynthate export, specific root exudate components, and selective microbial recruitment. Therefore, Si-mediated changes in carbon source availability are better interpreted as part of rhizosphere metabolic reprogramming rather than as a universal response across all plant–microbe systems. Field and multi-omics studies further indicate that Si can reshape rhizosphere niches under specific crop and soil conditions. For example, in a sugarcane field experiment, Si treatment increased rhizosphere available Si and selected nutrient contents, altered soil enzyme activities, and significantly restructured bacterial community composition and co-occurrence networks (Deng et al., 2021). Subsequent multi-omics analysis showed that these community shifts were strongly associated with changes in the concentrations of rhizosphere organic acids, sugars, and aromatic metabolites, suggesting that Si may shape a microenvironment favorable for colonization through the combined effects of root exudate metabolism and soil factors (Yuan et al., 2022). The extent to which this pattern applies to other soil textures, climatic regions, crop genotypes, and inoculant types is likely to depend on local edaphic and biological contexts.

Overall, Si-mediated regulation of the rhizosphere environment links root development, cell wall and cortical properties, rhizosphere chemistry, and metabolite availability with microbial colonization and functional maintenance. Its field performance is jointly influenced by soil buffering capacity, water fluctuations, salinity, heavy-metal toxicity, temperature variation, and competition from indigenous microbial communities. Therefore, the long-term functional significance of this process cannot be inferred solely from short-term changes in microbial abundance. Instead, it should be evaluated using integrated evidence from microbial dynamic tracking, root exudate profiling, soil available Si measurements, and assessments of microbial functional traits.

### 5.2 Si supports the functional expression and metabolic activity of beneficial microorganisms

Under severe stress conditions, even when microorganisms successfully colonize the rhizosphere, their functional capacities—such as nitrogen fixation, phosphate solubilization, and hormonal regulation—may still be suppressed due to the harsh environment. At this level, the role of silicon is primarily manifested in buffering stress in the host plant and rhizosphere environment, thereby indirectly maintaining microbial metabolic activity and functional expression, rather than directly enhancing the intrinsic functions of microbial strains. In sugarcane and winter wheat, Si application has been shown to improve carbon and nitrogen use efficiency, photosynthetic performance, and plant biomass (Wuyts et al., 2012; Frazão et al., 2020). These host-level improvements may increase the availability of carbon sources and nutrient resources in the rhizosphere, thereby providing a more stable environmental basis for maintaining microbial functions under stress conditions.

In legume–rhizobium symbiotic systems, Si may support nodulation and biological nitrogen fixation primarily through host-mediated stress buffering. For example, in Medicago truncatula, Si supply increased nodule number, nitrogenase activity, nitrogen accumulation, and biomass (Putra et al., 2022b), indicating that legumes may still benefit physiologically from Si treatment even though their Si accumulation is lower than that of typical high-Si-accumulating grasses. Consistent with this interpretation, recent evidence suggests that Si and SiO₂ nanoparticles may influence legume–rhizobia symbiosis by modulating nodulation, microbial metabolism, symbiotic signaling, infection-related processes, and oxidative stress responses, thereby supporting biological nitrogen fixation efficiency (Abd-Alla et al., 2026).

From the perspective of molecular mechanisms, although nod gene transcription in rhizobia is classically induced by plant-derived flavonoids, SiNP-induced changes in host metabolism or root exudation profiles may indirectly affect the activation of nod genes and subsequent Nod factor-mediated infection processes (Breakspear et al., 2014). (Dong and Song, 2020; Putra et al., 2022c)However, direct evidence demonstrating SiNP-mediated transcriptional activation of rhizobial nodABC genes remains limited, and the response is likely to be highly dependent on nanoparticle dose, plant genotype, rhizobial strain, and environmental context.Unlike the more evident tissue Si deposition and structural barrier reinforcement observed in high-Si-accumulating plants, the beneficial effects of Si in legumes may be manifested more through indirect buffering of stress status, including improved plant water relations, maintenance of redox homeostasis, enhanced antioxidant defense, regulation of nutrient uptake, and modification of the rhizosphere microenvironment (Johnson et al., 2018; El Moukhtari et al., 2022; Putra et al., 2022d). These changes may help alleviate the inhibitory effects of salinity, drought, or acid stress on root growth, nodule formation, and nodule function, thereby providing more favorable host physiological conditions for rhizobial infection, nodule development, and biological nitrogen fixation.

The relationship between Si and nutrient status, particularly phosphorus and potassium, may further influence nodulation and biological nitrogen fixation. Phosphorus is an important limiting factor for nodule development and nitrogen-fixing metabolism because nitrogen fixation in nodules requires high energy input, whereas P deficiency commonly suppresses nodule development, nitrogenase activity, and host nitrogen accumulation (Ma and Chen, 2021). Si treatment may indirectly affect P availability and uptake by influencing root growth, rhizosphere chemistry, and microbially mediated nutrient mobilization; however, this mechanism requires further validation across different legume crops and soil backgrounds. Potassium is involved in maintaining cellular turgor, stomatal regulation, enzyme activity, osmotic adjustment, and ionic balance. Particularly under salinity and drought stress, a higher K^+^/Na^+^ ratio and more stable plant water status may help mitigate stress-induced damage to nodule structure and nitrogen-fixing function (Liu et al., 2022). Recent ionomic and proteomic studies have also shown that Si supply can increase the concentrations of macronutrients such as P and K in nodules, accompanied by increases in nodule number, nitrogenase abundance, and plant nitrogen accumulation (Coquerel et al., 2024). Therefore, Si may indirectly support nodulation and biological nitrogen fixation in legumes by improving host stress-buffering capacity and influencing nutrient status, including P and K. However, this effect should still be interpreted conditionally in relation to Si source type, soil nutrient background, rhizobial strain, and stress type.

At the molecular level, the influence of Si on rhizobial signaling is more appropriately understood from a host-mediated perspective. The transcription of rhizobial nod genes is typically induced by plant-derived flavonoids and related root signals. Si or SiO₂ nanoparticles may indirectly affect nod gene activation and Nod factor-mediated infection by altering host metabolism, oxidative status, or root exudate composition (Breakspear et al., 2014) (Dong and Song, 2020; Putra et al., 2022c). At present, evidence that Si nanoparticles directly transcriptionally activate rhizobial nodABC genes remains limited, and this response may be influenced by nanoparticle dosage, plant genotype, rhizobial strain, and environmental context.

For PGPR and AMF systems, Si-mediated stress buffering may help maintain microbial functions by reducing host stress intensity and improving the rhizosphere environment. In a salt-stressed melon system, the combined application of Si and a PGPR consortium composed of Agrobacterium sp. B58, Pantoea sp. GR168, and Priestia sp. GR-1 increased seedling biomass and stem diameter while improving rhizosphere available nitrogen, available phosphorus, catalase activity, and acid phosphatase activity (Guo et al., 2024a). This suggests that Si–PGPR interactions may support plant performance partly by coordinating rhizosphere nutrient status and enzyme activities. In an AMF system, the combined application of soluble Si and AMF inoculation increased dry matter accumulation, AMF colonization, and essential ion balance in salt-stressed baby corn, particularly through increases in K^+^, Ca^2+^, and the K^+^/Na^+^ ratio (Islam et al., 2023a). These examples support the view that Si does not necessarily directly stimulate microbial metabolism, but may instead create a plant–rhizosphere environment with reduced stress intensity, thereby facilitating the maintenance of PGPR and AMF functions.

Si-mediated stress buffering may also affect other functional microorganisms. For example, fungi in the genus Trichoderma may benefit from improved host root physiological status or changes in root exudates (Fan et al., 2016b; Wang et al., 2021b). Salinity stress can also induce plants to recruit specific rhizosphere and endophytic bacterial communities, thereby enhancing adaptation to salt stress (Li et al., 2021). Cyanobacteria, as carbon- and nitrogen-fixing microorganisms, can contribute to improvements in soil fertility, organic carbon, and nitrogen status (Ramakrishnan et al., 2023). Archaea may respond to salt stress-induced carbon limitation by enriching genes related to salt tolerance, metabolism, and carbon acquisition (Dong et al., 2024). However, whether cyanobacteria and archaea participate in Si-mediated regulation of plant stress tolerance remains unsupported by direct evidence.

Studies related to nanomaterials also support the stress-buffering role of Si, although their interpretation must be context-specific. Under Cd contamination, SiO₂ nanoparticles reduced Cd accumulation in the roots and leaves of bayberry, enhanced antioxidant capacity, and shifted the rhizosphere microbial community toward functional groups associated with detoxification and nutrient cycling (Ahmed et al., 2024). Under drought stress, SiO₂ nanoparticles increased antioxidant enzyme activities in Ehretia macrophylla seedlings and regulated stress-related metabolic pathways (Chen et al., 2024). These studies indicate that Si-based materials may help maintain microbial functions by reducing host stress intensity and modifying the rhizosphere environment. However, their effects are influenced by particle properties, dosage, exposure duration, and soil background.

Overall, Si-mediated stress buffering may maintain microbial functional activity by stabilizing plant physiological status, carbon source supply, nutrient balance, and rhizosphere redox conditions. Future studies need to distinguish among direct microbial responses, host-mediated effects, and nanoparticle-specific effects. They should also further evaluate whether key microbial functional modules, including ACC deaminase activity, IAA production, siderophore synthesis, EPS formation, and nitrogen fixation, exhibit consistent responses across different crops, soil types, and stress conditions that approximate field reality.

### 5.3 Activation of rhizosphere Si by beneficial microorganisms

A major limitation of conventional Si fertilization is that part of the applied Si is readily adsorbed, precipitated, or fixed after entering the soil. The limited bioavailability of H₄SiO₄ is therefore one of the key reasons for the low use efficiency of Si fertilizers. Beneficial microorganisms, particularly silicate-solubilizing bacteria (SSB), phosphate-solubilizing microorganisms with silicate-weathering capacity, and some PGPR, may partially overcome this limitation by promoting the release of mineral-bound Si into plant-available H₄SiO₄.

Microbial activation of Si is mainly associated with organic acid secretion, EPS, enzymatic activities, and mineral weathering processes. Low-molecular-weight organic acids, such as citric acid and oxalic acid, may lower rhizosphere pH, chelate mineral-bound cations, and promote the dissolution of aluminosilicate minerals. For example, the Rhizobium strain IIRR-1 isolated from the rice rhizosphere showed substantial silicate-solubilizing capacity. In media containing poorly soluble Si sources such as feldspar and diatomite, this strain significantly increased dissolved Si concentrations and, in pot experiments, enhanced rice biomass and plant Si content (Chandrakala et al., 2019). Similarly, several silicate-solubilizing bacteria, including Bacillus and Pseudomonas, have been reported to accelerate the weathering of aluminosilicates such as feldspar and quartz, increase dissolved Si levels, and often simultaneously improve P and K availability (Vasanthi et al., 2018; Raturi et al., 2021). These findings indicate that microbial Si activation is often linked to broader nutrient-cycling processes rather than representing an isolated Si-specific process.

Phosphate-solubilizing microorganisms may also contribute to enhanced Si availability. In pot experiments with wheat (Triticum aestivum L.), the combined application of phosphate-solubilizing bacteria and Si increased soil available P, altered the proportions of different soil Si fractions, expanded the available Si pool, and was accompanied by simultaneous increases in shoot Si and P contents. These results suggest that some phosphate-solubilizing bacteria may also have the potential to mobilize Si (Rezakhani et al., 2019). Other studies have also indicated that Si-solubilizing bacteria are widely present across different soil types, but their solubilization efficiency and persistence are strongly influenced by pH, mineral composition, and competition with indigenous microbial communities (Etesami and Schaller, 2023). This suggests that some microorganisms traditionally classified as phosphate solubilizers may also affect Si activation through rhizosphere acidification and mineral transformation. However, this effect is likely to depend on soil mineral composition and the solubility of the applied Si source.

Therefore, differences among Si sources are critical for interpreting Si–microorganism interactions. Soluble silicates, calcium silicate, potassium silicate, sodium silicate, diatomite, feldspar, biogenic Si, and SiO₂ nanoparticles differ in dissolution rate, accompanying cations, pH effects, and interactions with soil colloids. Consequently, plant growth improvement following combined Si–microorganism treatments should be interpreted together with evidence such as soil available Si, plant Si content, Si deposition, or Si transporter expression. For Si sources containing accompanying cations, such as potassium silicate, sodium silicate, or calcium silicate, future experiments should include ion-balanced and pH-balanced controls to distinguish Si-specific effects from K^+^, Na^+^, or Ca^2+^ effects, as well as alkalinity- or pH-mediated effects. For example, depending on the composition of the Si source, KCl, NaCl, CaCO₃, or corresponding pH-adjusted treatments could be included as controls to avoid incorrectly attributing the effects of accompanying cations or soil chemical changes to Si–microorganism synergy.

Overall, microbial activation of mineral-bound Si is one of the key mechanisms that distinguishes Si–microorganism synergy from Si application or microbial inoculation alone. However, the field relevance of this mechanism depends on whether functional strains can persist and remain active under salinity, drought, heavy-metal toxicity, extreme pH, and competition from indigenous microorganisms. Future studies should link microbial Si-solubilizing traits with the dynamics of soil Si fractions, plant Si uptake, and stress-tolerance physiological responses, and validate these relationships across different soil types and experimental scales.

### 5.4 Beneficial microorganisms influence plant Si uptake and internal distribution through rhizosphere regulation and transport-related processes

After Si has been mobilized and becomes available in soluble forms, its stress-mitigating effects still depend on root uptake and internal distribution within the plant. Beneficial microorganisms may influence these processes through both soil-side and plant-side mechanisms, including promotion of root growth, enhancement of nutrient availability, and regulation of Si transport-related pathways. However, the relative contribution of these mechanisms varies with the Si accumulation capacity of the crop and should therefore be evaluated using direct Si-related evidence.

The rice–AMF system provides one of the clearest examples of microbial involvement in Si acquisition. Inoculation with Rhizophagus irregularis can increase shoot Si accumulation in rice. Stable-isotope tracing and hyphal-compartment experiments further indicate that AMF hyphae can participate in Si uptake and transfer Si to the host. Meanwhile, the expression of Si transporter genes such as *Lsi1* and *Lsi2* is upregulated in roots, suggesting that fungal uptake pathways and the plant endogenous transport system may act synergistically during Si acquisition (Qiu et al., 2024a).This interpretation is consistent with the recently proposed “soil silicon filter” concept, which suggests that mycorrhizal fungi and their associated microbiome may collectively regulate biosilicification, microbial Si transformation, and plant-available Si flux at the mycorrhizosphere interface (Etesami and Schaller, 2026). Because Lsi1 is a passive NIP-type aquaporin that mediates H₄SiO₄ influx, whereas Lsi2 is an active efflux transporter involved in radial transport and xylem loading, microbial effects on these transport processes may directly alter Si acquisition efficiency in crops with functional Si transport modules.

In non-rice systems, microbial effects on Si uptake may rely more heavily on indirect processes. In wheat grown in calcareous soil, the combined application of Si fertilizer and phosphate-solubilizing bacteria not only increased plant uptake of Si and P but also altered the proportions of different soil Si fractions, thereby expanding the available Si pool (Rezakhani et al., 2022). In addition, rhizosphere strains with silicate-solubilizing capacity, IAA production, and ACC deaminase activity can promote root length, lateral root formation, and root hair development, thereby increasing the root absorptive surface area (Chandrakala et al., 2019). These processes provide plausible pathways through which microbial activity may promote Si acquisition, particularly when increases in plant Si concentration, Si deposition, Si flux, or Lsi expression are observed simultaneously.

Therefore, crop Si accumulation capacity is a critical factor when interpreting microbially promoted Si uptake. In high-Si-accumulating plants such as rice, microbial enhancement of Si availability may be more readily coupled with active root uptake and xylem loading. By contrast, in species or genotypes with low Si accumulation capacity, the benefits of combined Si–microorganism application may arise more from improved rhizosphere conditions, enhanced root growth, nutrient balance, or stress signal regulation, rather than necessarily being reflected in a substantial increase in tissue Si concentration.

Overall, beneficial microorganisms may promote Si uptake and internal distribution by linking Si dissolution, root morphological regulation, rhizosphere nutrient dynamics, and transport-related processes. Future studies should simultaneously quantify soil available Si, dissolved H₄SiO₄, plant Si concentration, Si deposition patterns, and Lsi expression, and should assess whether microbial inoculation alters Si allocation among plant organs under different stress conditions. Such an evidence chain would help distinguish whether combined treatments improve plant performance by enhancing Si uptake, modifying internal Si distribution, or primarily acting through rhizosphere-mediated and physiological effects.

### 5.5 Stress-specific coordination of plant physiological defense by combined Si–microorganism application

The physiological effects of combined Si and beneficial microorganism application are commonly manifested as improved plant growth, reduced oxidative damage, optimized ionic or nutrient balance, and alleviated stress injury. However, these responses should not be interpreted as the outcome of a single universal mechanism, as they may depend on stress type, plant species, Si source properties, and other contextual factors (Cooke and Leishman, 2016). Taking salinity stress as an example, relatively direct evidence from both field and controlled-condition studies supports the synergistic effects of Si and beneficial microorganisms. In a salt-stressed mung bean field system, PGPR inoculation combined with foliar Si application improved plant water relations, photosynthetic pigment accumulation, growth, and grain yield (Mahmood et al., 2016b). Subsequent studies further showed that the combined application of Bacillus drentensis or Enterobacter cloacae with foliar Si enhanced antioxidant enzyme activities, reduced lipid peroxidation, and improved mineral nutrient uptake and yield performance in mung bean (Mahmood et al., 2022a). Similarly, in salt-stressed melon and baby corn systems, Si–PGPR or Si–AMF combined treatments have been reported to improve rhizosphere nutrient status, soil enzyme activities, mycorrhizal colonization, and K^+^/Na^+^ balance (Islam et al., 2023b; Guo et al., 2024b).

It should be noted that even under salinity stress, the dominant mechanisms may differ among crops and Si sources. In high-Si-accumulating crops such as rice, Si–beneficial microorganism interactions may be more readily coupled with the plant’s endogenous Si uptake and transport processes. For example, bacterial inoculants combined with nano-Si improved growth, antioxidant status, and yield performance in salt-stressed rice, whereas AMF inoculation was also shown to increase shoot Si accumulation and upregulate Si transport-related genes in rice roots (Alharbi et al., 2022b; Qiu et al., 2024b). By contrast, in non-rice systems such as melon, sugar beet, and legumes, the benefits of combined treatment may depend more on rhizosphere nutrient availability, antioxidant metabolism, and the functional traits of the inoculants, rather than on high levels of tissue Si deposition. For example, an Si–PGPR consortium increased rhizosphere available N/P and enzyme activities in melon (Guo et al., 2024c); PGPR combined with SiO₂ nanoparticles improved redox status and K^+^/Na^+^ balance in sugar beet (Alharbi et al., 2022a); and PGPR inoculation combined with foliar Si improved water relations, photosynthetic pigment accumulation, growth, and grain yield in mung bean (Mahmood et al., 2016c). In addition, different Si sources vary in H₄SiO₄ release rate, accompanying cation composition, pH-regulating capacity, and particle behavior. Soluble silicates may rapidly increase available Si levels and alter pH conditions in the system (Thakral et al., 2024); potassium silicate or calcium silicate may also introduce K^+^ or Ca^2+^ related effects (Schaller et al., 2024; Yuan et al., 2024); and the effects of SiO₂ nanoparticles may be influenced by particle size, surface properties, dissolution and release behavior, and rhizosphere accumulation (Wang et al., 2022b; Pan et al., 2023). Therefore, plant defense responses induced by different Si sources should not be directly equated with Si-specific effects; rather, they need to be distinguished using appropriate controls for pH, accompanying ions, and particle-related effects.

Compared with salinity stress, direct evidence for the combined use of Si and beneficial microorganisms in alleviating drought stress remains relatively limited. Current explanations rely more on the overlap between Si-mediated drought tolerance mechanisms and microbe-mediated plant growth-promoting mechanisms under drought. Under drought conditions, Si can alleviate water deficit-induced damage by improving plant water relations, maintaining photosynthetic function, regulating osmotic balance, and enhancing antioxidant defense (Wang et al., 2021c). AMF hyphal networks can improve water supply at the soil–root interface by increasing soil water conductivity, extending effective root length, and buffering the decline in rhizosphere matrix potential (Abdalla et al., 2023). PGPR can also participate in drought responses by regulating root morphology, producing phytohormones and osmoprotectants, forming extracellular polymers, and enhancing ACC deaminase activity and antioxidant-related functions (Ahmad et al., 2022). Recent case studies further suggest that SiO₂ nanoparticles combined with plant growth-promoting bacteria (PGPB) can improve biomass, N/P/K/Si uptake, MDA levels, and antioxidant enzyme activities in wheat under water deficit (Davoudi et al., 2024). Combined application of Si, PGPR, and rhizobia has also been shown to enhance photosynthesis-related parameters, compatible solute accumulation, ion content, and antioxidant responses in chickpea under water deficit (Kamal et al., 2025b). However, these studies are still largely derived from specific crops and controlled environmental conditions. They are not yet sufficient to demonstrate that Si and beneficial microorganisms generally form stable synergy under drought stress through shared signaling networks, nor can they exclude the possibility that plant water status is improved mainly through the additive effects of their respective pathways. Therefore, Si–microorganism synergy under drought conditions should be validated by integrating evidence on root hydraulic traits, rhizosphere water dynamics, AMF hyphal contribution, inoculant colonization and functional expression, Si deposition patterns, and antioxidant responses, rather than being inferred solely from biomass or a single antioxidant enzyme activity indicator.

Under heavy-metal or metalloid stress, interpretation of Si–beneficial microorganism interactions should move beyond the outcome of “reduced metal accumulation” and further distinguish among metal immobilization, uptake restriction, transport inhibition, metabolic detoxification, and microbially mediated rhizosphere regulation. Recent studies on nano-Si and foliar nanoparticles provide mechanistically informative examples. For instance, foliar application of Si nanoparticles (SiNPs) induced coordinated defense against Cd in rice through jasmonic acid-mediated metabolic reprogramming, indicating that Si-based materials may alleviate Cd stress not only through physical blocking or rhizosphere immobilization but also through systemic regulation of hormone signaling, metabolic networks, and defense responses (Li et al., 2026). Another study in wheat showed that foliar application of nanoparticles, including SiO₂ nanoparticles, reduced grain Cd content through long-distance “leaf–root–microorganism” regulation. This suggests that aboveground nanoparticle treatments may alter metal transport to grains by affecting root processes, rhizosphere microorganisms, and Cd bioavailability (Wang et al., 2024). These studies cannot be directly equated with Si–beneficial microorganism systems involving exogenous PGPR or AMF inoculation. Nevertheless, they indicate that the effects of Si-related materials under metal stress may be jointly mediated by plant signaling, rhizosphere metabolism, and microbiome shifts.

Consistent with this view, previous studies on Si physiology have shown that Si-mediated alleviation of metal or metalloid toxicity may involve rhizosphere metal immobilization, restriction of root uptake, cell wall binding, apoplastic retention, subcellular sequestration, and regulation of the antioxidant system (Vaculík et al., 2020) (Hou et al., 2023). Beneficial microorganisms such as PGPR and AMF can alter rhizosphere metal speciation and bioavailability through EPS, biofilm formation, organic acids, siderophores, extracellular complexation, and biosorption (Hnini et al., 2024). Recent studies on foliar nanoparticles and Si-NP–PGPR combinations further suggest that reduced Cd accumulation may be jointly associated with long-distance “leaf–root–microorganism” regulation, decreased soil Cd bioavailability, and restricted root–stem–grain transport (Alharbi et al., 2024). Therefore, in Si–microorganism combined systems, a decrease in metal accumulation within plants does not automatically indicate that a single pathway is dominant. Future studies should incorporate soil metal speciation, rhizosphere complexing or adsorptive components such as organic acids, siderophores, and EPS, root–stem–grain translocation factors, subcellular metal distribution, plant hormone and metabolic reprogramming features, and microbial metal-tolerance traits into a unified evidence chain to clarify the actual contribution of Si–microorganism interactions to metal stress alleviation.

Therefore, the key question in Si–beneficial microorganism-mediated stress tolerance is not merely whether a given physiological indicator is improved, but whether the Si source, inoculant function, crop Si accumulation capacity, and specific stress combination can jointly support stable matching of defense modules. Future research should move from single-indicator physiological evaluation toward multi-layer evidence integration. Si uptake and deposition, ionic homeostasis, rhizosphere metabolites, inoculant colonization and functional genes, redox status, and yield stability should be incorporated into the same analytical framework to determine the dominant mechanisms and applicability boundaries of Si–microorganism synergy under different stress contexts.

## 6 Perspectives

### 6.1 Elucidating the molecular communication mechanisms in the “silicon–beneficial microorganism–plant” tripartite interaction

Although silicon and beneficial microorganisms exhibit clear synergistic effects in enhancing plant tolerance to drought, salinity, and oxidative stress, it remains unclear whether they operate through relatively independent regulatory pathways or achieve coordinated regulation via shared and interconnected signaling networks within plants (Verma et al., 2020; Rajput et al., 2021; Etesami, 2025). Therefore, future research should shift from single-factor studies toward a systems-level analysis of the multi-component interaction network among silicon, microorganisms, and plants.

**Figure 4:**
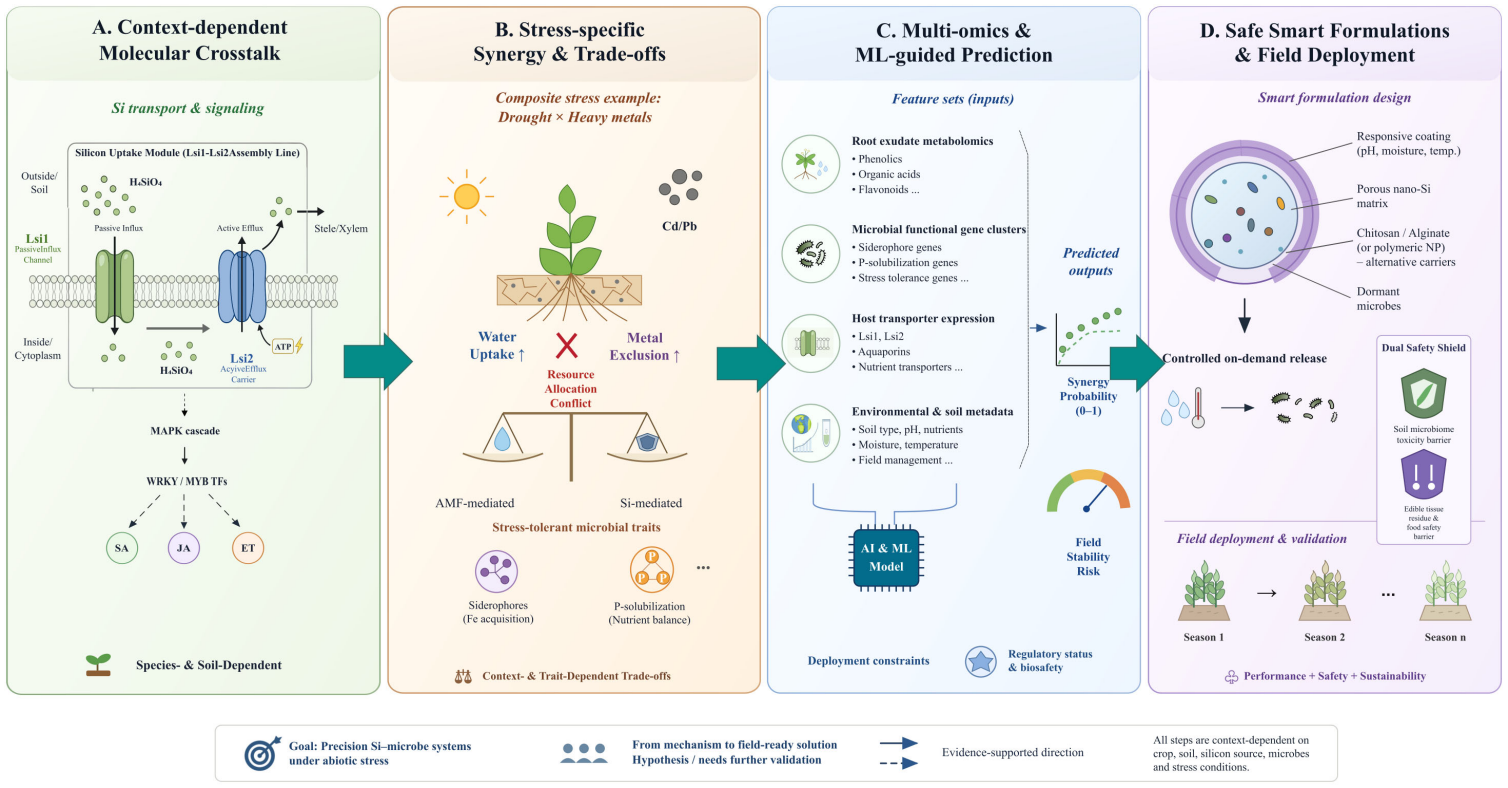
Conceptual framework for future research on the synergistic regulation of plant abiotic stress tolerance by silicon and beneficial microorganisms. **(A)** Putative molecular communication potentially involving crosstalk between silicon transport processes and microbe-induced systemic resistance (ISR) signaling pathways, including MAPK cascades and SA/JA/ET-related signaling networks. **(B)** Context-dependent functional trade-offs under combined abiotic stresses (e.g., drought and heavy metal exposure), potentially involving coordination between AMF-associated water acquisition and Si-related regulation of ion sequestration and compartmentalization. **(C)** Prospective development of silicon-based bioformulations through multi-omics integration and machine learning approaches for optimized and site-specific field applications. **(D)** Emerging strategies for stimuli-responsive nano-carriers enabling controlled or “on-demand” release of microbial inoculants, with potential implications for improving microbial survival and formulation efficiency. MAPK: Mitogen-activated protein kinase; ROS: reactive oxygen species; TF: transcription factor; SA: salicylic acid; JA: jasmonic acid; ET: ethylene; PGPR: plant growth-promoting rhizobacteria; AMF: arbuscular mycorrhizal fungi; Cd/Pb: cadmium/lead; P-solubilization: phosphate solubilization; AI: artificial intelligence.

At the molecular level, the potential interaction between silicon signaling and microbe-induced systemic resistance (ISR) remains largely unexplored. As illustrated in **Figure 4A**, it is still unclear whether silicon uptake and transport mediated by silicon transporters (e.g., Lsi1 and Lsi2) participate in the regulation of MAPK signaling pathways, reactive oxygen species (ROS) scavenging systems, and hormone signaling networks involving salicylic acid (SA), jasmonic acid (JA), and ethylene, and whether these processes synergize with microbe-induced defense responses (Khan et al., 2023; Thakur et al., 2023; Qiu et al., 2024a). In this context, silicon may function not only as a structural or nutritional component but also as a regulatory factor within plant signaling networks. For instance, it may modulate the expression or activity of specific transcription factors, such as members of the WRKY or MYB families, thereby participating in plant stress responses and plant–microbe interactions. Furthermore, silicon may act as a signal modulator or amplifier, enabling plants to more rapidly activate ISR or related defense responses under low-intensity stress or microbial stimuli by enhancing MAPK cascades or hormone signaling pathways. Verification of this potential mechanism would provide important insights into the molecular basis of the synergistic effects between silicon and beneficial microorganisms.

In addition, whether silicon can regulate the rhizosphere environment by altering root exudate composition, thereby influencing the colonization of beneficial microorganisms on root surfaces, remains to be clarified. Plants are known to actively shape rhizosphere microbial community structure by adjusting the composition and concentration of root exudates under different nutritional and stress conditions (Tao et al., 2024). Thus, silicon may influence the release patterns of organic acids, amino acids, and phenolic compounds by modulating plant carbon metabolism or secondary metabolic pathways.

It is noteworthy that similar plant–microbe synergistic regulatory mechanisms have been well characterized in other nutrient systems. For example, in carbon-driven rhizosphere interactions, plants actively regulate microbial community structure by releasing sugars, organic acids, and secondary metabolites, thereby promoting the colonization and functional expression of beneficial microorganisms (Mashabela et al., 2022; Wu et al., 2025). In nitrogen and phosphorus cycling systems, plants establish stable symbiotic or associative relationships with rhizobia or phosphate-solubilizing bacteria through specific signaling molecules, such as flavonoids, thereby promoting nutrient acquisition and plant growth (Singh et al., 2022; Li et al., 2024). Specifically, in the phosphorus–phosphate-solubilizing bacteria–arbuscular mycorrhizal fungi (P–PSB–AMF) system, AMF can channel plant-derived photosynthetic carbon belowground and promote the growth and activity of phosphate-solubilizing bacteria (PSB) through hyphal exudates. In turn, PSB can enhance the phosphorus-acquisition capacity of AMF by promoting organic P mineralization. However, this AMF–PSB mutualism is regulated by background soil P availability: under low-P conditions, AMF and PSB may compete for P resources, whereas under relatively sufficient P supply, they tend to exhibit mutually reinforcing effects (Zhang et al., 2014, Zhang et al., 2016; Etesami et al., 2021). The Fe–siderophore system mainly centers on microbial siderophore-mediated Fe acquisition. Under Fe-limited conditions, microorganisms can secrete high-affinity Fe-chelating compounds, namely siderophores, to promote the complexation, dissolution, and cellular uptake of poorly soluble or weakly available Fe (Neilands, 1995; Hider and Kong, 2010; Schalk, 2025a). Meanwhile, because some siderophores can also form complexes with various non-Fe metals, this process may further alter metal speciation, mobility, and rhizosphere availability in contaminated soils or soils with limited metal availability (Neubauer et al., 2000; Schalk, 2025b).

In nitrogen and phosphorus cycling systems, plants establish stable symbiotic relationships with rhizobia or phosphate-solubilizing bacteria through specific signaling molecules, such as flavonoids, to enhance nutrient acquisition and plant growth (Singh et al., 2022; Li et al., 2024). These findings suggest that nutrients are not only essential components of plant metabolism but may also play regulatory roles in plant–microbe interactions. Drawing on these frameworks, it is plausible that silicon may similarly regulate rhizosphere signaling exchange, microbial recruitment, or symbiotic efficiency, thereby contributing to plant stress resistance.

Within symbiotic systems, it remains unclear whether silicon can enhance nodulation efficiency or promote mycorrhizal hyphal expansion by influencing cell wall structure, carbon allocation, or energy metabolism. For instance, silicon deposition in plant cell walls may alter their mechanical properties or porosity, thereby affecting the infection and colonization processes of symbiotic microorganisms within root tissues. Additionally, silicon-mediated improvements in photosynthetic efficiency and carbon assimilation may influence carbon allocation to the rhizosphere or symbiotic structures, such as nodules or mycorrhizae, thereby affecting the stability of these associations. However, these potential mechanisms currently lack direct molecular and ecological evidence.

### 6.2 Synergistic enhancement of tolerance to combined stresses and nutrient use efficiency

In natural environments, plants are typically exposed to multiple simultaneous environmental stresses, often accompanied by limited soil nutrient availability. However, current research on the synergistic effects of silicon and beneficial microorganisms has largely focused on single-stress scenarios, and the coupling mechanisms between stress resistance and nutrient acquisition under combined stress conditions remain poorly understood (Verma et al., 2020). Therefore, future studies should move beyond single-stress models toward more complex stress systems that better reflect natural conditions, in order to systematically elucidate the coordinated roles of silicon and beneficial microorganisms in regulating plant stress tolerance and nutrient use efficiency.

In terms of nutrient acquisition, silicon may facilitate the mobilization of insoluble mineral nutrients by influencing rhizosphere chemical conditions and plant–microbe signaling interactions. Many PGPR and mycorrhizal fungi enhance the solubilization of poorly available phosphorus through the secretion of organic acids or phosphatases, while also improving iron bioavailability via the production of siderophores that chelate and transport Fe^3+^ (Wang et al., 2021a; Pang et al., 2024; Yang et al., 2025b). In this context, it remains to be clarified whether silicon can modulate the composition or concentration of root exudates—such as organic acids, phenolic compounds, or sugars—thereby altering rhizosphere pH or chemical signaling environments and subsequently influencing the metabolic activity of phosphate-solubilizing or siderophore-producing microorganisms (Rajput et al., 2021). Similar rhizosphere chemical regulation mechanisms have been widely documented in plant–microbe interaction studies (Tao et al., 2024). Thus, whether silicon indirectly participates in this process through the regulation of plant carbon metabolism or secondary metabolism requires further investigation.

In addition, within AMF symbioses, hyphal networks significantly expand the effective absorptive area of plant roots and facilitate the transport of water and mineral nutrients, thereby enhancing plant adaptation under drought and salinity stress (Hajiboland et al., 2018; Qiu et al., 2024a). At the same time, AMF hyphae and associated structures can adsorb or immobilize heavy metal ions, reducing their translocation to aboveground tissues and providing an important ecological buffering function in contaminated environments (Ahmed et al., 2026). However, under combined stress conditions, potential trade-offs may arise among these functions, as illustrated in **Figure 4B**. For example, in environments characterized by both drought and heavy metal contamination, the AMF-mediated enhancement of water acquisition may coexist with the need to restrict the upward transport of toxic metals (Wang et al., 2022a).

In this context, it remains unclear whether silicon can simultaneously promote water uptake while limiting heavy metal translocation by regulating apoplastic barriers, enhancing cell wall immobilization capacity, or modifying ion transport pathways. A deeper understanding of this functional balance between “water transport” and “metal sequestration” will be essential for elucidating the ecological mechanisms underlying silicon–microbe synergistic interactions under complex stress conditions.

### 6.3 Precision development driven by multi-omics technologies and digital modeling

With the advancement of multi-omics technologies, research on the synergistic stress resistance of silicon and microorganisms is shifting from physiological characterization toward mechanistic dissection at the molecular level. Mahmood et al (Mahmood et al., 2017). demonstrated that the combined application of silicon and specific PGPR strains (e.g., Enterobacter cloacae) significantly improved ionic balance in mung bean, thereby alleviating salt stress and increasing crop yield. Subsequent studies suggest that this synergistic effect may be achieved through both signaling regulation and metabolic adjustment. On the one hand, PGPR reduce the accumulation of ethylene precursors in plants by secreting ACC deaminase, thereby alleviating stress-induced growth inhibition (Gupta et al., 2022). On the other hand, the combined application of silicon and PGPR promotes the accumulation of osmolytes such as proline and enhances the activity of antioxidant enzymes including superoxide dismutase (SOD) and catalase (CAT), thereby improving the capacity of plants to maintain cellular homeostasis under abiotic stresses such as salinity and drought (Zhao et al., 2025).

However, systematic understanding of the spatial specificity and signal interaction mechanisms underlying this synergistic regulatory network remains limited. Plant roots consist of multiple cell types—including epidermis, cortex, endodermis, and stele—each playing distinct roles in transport processes, signal perception, and microbial colonization. Yet most current studies rely on omics analyses at the whole-plant or whole-root level, which limits the resolution needed to distinguish functional differences among cell types in plant–microbe interactions (Verbon et al., 2023). Furthermore, although plants can regulate rhizosphere microbial community structure through the secretion of organic acids, sugars, and secondary metabolites—for example, enhancing phosphate-solubilizing microbial colonization under phosphorus deficiency—direct evidence is still lacking as to whether silicon-induced changes in root exudates can enable the targeted recruitment of specific beneficial microorganisms, such as phosphate-solubilizing or siderophore-producing microbes, within silicon–microbe synergistic systems.In terms of multi-omics feature selection, future studies should move beyond descriptive omics profiling toward quantifiable sets of functional traits. For example, priority could be given to integrating root exudate metabolomic features, including organic acids, sugars, amino acids, phenolic acids, and flavonoid signaling molecules; microbial functional traits, including siderophore biosynthesis, organic acid secretion, EPS formation, ACC deaminase activity, and gene clusters related to silicate solubilization; and host-side indicators of Si uptake and stress responses, including Lsi1/Lsi2 expression, plant Si content, Si deposition patterns, antioxidant enzyme activities, the K^+^/Na^+^ ratio, and yield stability. Together, these features can establish a multi-layer evidence chain for explaining and predicting Si–microorganism synergistic effects.

Based on these observations, we propose that silicon deposition in root cell walls and apoplastic spaces may alter local ionic environments and redox status, thereby influencing plant perception of microbial signaling molecules and potentially amplifying defense or stress-resistance signaling pathways induced by PGPR. Validation of this hypothesis would provide a new theoretical framework for understanding silicon–microbe synergistic interactions.

Beyond cell-level mechanistic insights, the integration of multi-omics data with digital modeling is transforming research paradigms in plant–microbe interactions. In recent years, the accumulation of large datasets on soil properties, climatic factors, and microbial community structures has enabled the application of machine learning algorithms to predict plant–microbe interaction patterns. For instance, by integrating soil physicochemical properties, climate data, and microbial diversity information, predictive models can be constructed to assess the stability of plant–microbe synergistic effects under different environmental conditions (Peng et al., 2022; Hagen et al., 2024). In the context of silicon–microbe synergistic applications, such models hold promise for predicting optimal silicon application rates and functional microbial combinations across diverse soil types and environmental conditions, thereby enabling the development of optimized application strategies. As illustrated in **Figure 4C**, machine learning models can further integrate multi-omics data, environmental variables, and crop growth parameters to predict microbial community responses at large scales and optimize the application of silicon-based bioformulations. This approach will facilitate the transition from empirical application toward precision design in silicon–microbe synergistic technologies, ultimately enhancing their stability and adaptability in agricultural production (Mo et al., 2024).In terms of modeling objectives, machine-learning models should not be used only to screen optimal Si sources or inoculant combinations. They can also be further developed to predict the probability and stability of Si–microorganism synergistic effects under specific soil types, crop species, stress backgrounds, and inoculant functional traits. Potential input variables may include soil pH, organic matter content, texture, available Si/P/K, salinity, heavy-metal bioavailability, crop Si accumulation type, root traits, microbial functional genes, and climatic factors. Output variables may include biomass, yield, antioxidant responses, nutrient uptake, inoculant persistence, and synergy scores. However, the application of such models is still constrained by insufficient standardized cross-site datasets, limited field validation, the regulatory status of nano-Si materials and microbial inoculants, formulation stability, and requirements for ecological safety assessment.

In addition, future studies should introduce quantitative metrics for evaluating synergistic effects to distinguish true synergy, additivity, and antagonism between Si and beneficial microorganism treatments. Most current studies infer synergy mainly by comparing phenotypic differences among Si application alone, microbial inoculation alone, and combined treatment. However, this approach cannot effectively exclude simple additive effects. The Bliss independence model can be used to estimate the expected additive effect under the assumption that two treatments act independently. The Highest Single Agent (HSA) model can assess whether a combined treatment outperforms either single treatment. The Loewe additivity model is suitable for datasets with dose–response relationships and can be used to determine whether a combined treatment exceeds the expected effect of equivalent dose additivity. In Si–microorganism research, these metrics can be applied to standardized endpoints such as biomass, yield, nodule number, nitrogenase activity, antioxidant enzyme activity, the K^+^/Na^+^ ratio, plant Si content, and soil available Si. Future experiments should include complete Si dose gradients, inoculant gradients, and combined-treatment matrices, and should report synergy scores together with confidence intervals to improve the comparability and reproducibility of Si–microorganism interaction effects across different crops, soils, and stress conditions.

Existing field evidence provides preliminary support for the agricultural relevance of combined Si and beneficial microorganism application, but its effects are clearly context-dependent. In a two-year field experiment with mung bean under saline-water irrigation, PGPR inoculation, particularly with Bacillus drentensis or Enterobacter cloacae, combined with foliar Si application improved plant water-related traits, photosynthetic pigments, antioxidant metabolism, nutrient uptake, and grain yield. These results suggest that PGPR–foliar Si combinations can improve specific physiological and yield-related traits under field salinity stress; however, current evidence is still largely concentrated in a limited number of crops and specific stress scenarios (Mahmood et al., 2016d, Mahmood et al., 2022b).

Other field or near-field studies provide complementary but still condition-dependent evidence. In faba bean grown in salt-affected soil, foliar potassium silicate combined with PGPR improved nodulation, root growth, K^+^/Na^+^ balance, water status, photosynthetic performance, nutrient uptake, and grain yield (Hafez et al., 2021b). However, because potassium silicate supplies both Si and K, these responses should be interpreted as combined-treatment effects rather than being directly attributed to Si-specific uptake in the absence of ion-balanced controls. In maize exposed to the combined effects of water deficit and soil salinity, PGPR combined with SiO₂ nanoparticles improved soil enzyme activities, plant physiological status, nutrient uptake, and yield-related traits. Nevertheless, nanoparticle-specific effects, microbial effects, and changes in soil physicochemical properties may have contributed simultaneously to these responses (Hafez et al., 2021c; Sharf-Eldin et al., 2023).

From an economic perspective, direct cost–benefit analyses of combined Si–microorganism application remain limited. Its feasibility may depend on Si source type, inoculant formulation, application frequency, labor or mechanization costs, yield gains, and stress-loss mitigation in high-risk production areas. Compared with nanoformulations, conventional silicate fertilizers combined with seed, soil, or foliar microbial inoculation may be more readily integrated into existing crop management systems. However, their profitability still needs to be verified through multi-site and multi-season field trials (Schütz et al., 2018; Su et al., 2022). Therefore, in addition to reporting biomass, yield, and physiological traits, future field studies should also record input costs, net returns, benefit–cost ratios, inoculant persistence, soil available Si dynamics, and environmental risk indicators.

### 6.4 Engineering exploration of innovative silicon–microbe composite formulations

Although Si and beneficial microorganisms exhibit significant synergistic effects in alleviating abiotic stress in plants, translating these findings into stable and scalable agricultural products remains a major challenge. Compared with conventional chemical fertilizers, microbial formulations often face practical limitations, including low survival rates of inoculated strains, poor environmental adaptability, and limited shelf life (Aloo et al., 2022). Therefore, future research should not only further elucidate the biological mechanisms underlying silicon–microbe interactions but also strengthen the integration of material engineering and formulation technologies to develop stable and efficient composite biofertilizer systems.

Nano-Si is considered a promising microbial carrier material because of its large specific surface area and porous structure. Studies have shown that nanomaterials can form stable microbial carriers through encapsulation or surface coating, thereby improving the survival of functional strains under environmental stress conditions and enabling the slow release of nutrients or functional factors (Kaur et al., 2023; Fernandes and Sharma, 2026). However, not all nano-Si materials are suitable as microbial carriers, as pore structure, surface charge, and chemical modification can all affect microbial adsorption and colonization efficiency on the carrier. For example, Si-based materials with appropriate pore size and surface hydrophilicity can provide a stable microenvironment for microorganisms, whereas chemical modification through the introduction of functional groups such as carboxyl and amino groups may further strengthen interfacial interactions between the carrier and microbial cell walls, thereby protecting strains while maintaining their metabolic activity (Izquierdo-Barba et al., 2011; Zakeri Siavashani et al., 2016; Rivas et al., 2023). In nanotechnology-enabled integrated microbial formulation design, future products should simultaneously consider carrier pore structure, surface charge, hydrophilicity, surface functional groups, strain compatibility, environmentally responsive release, and storage stability. In addition to porous nano-Si, chitosan, alginate, and other biodegradable polymers may also serve as protective coating materials or auxiliary carriers. These materials can be combined with Si-based materials to construct composite delivery systems that improve strain survival, release controllability, and field applicability.

In addition to Si-based nanocarriers, chitosan, alginate, and other biodegradable polymer nanoparticles can also serve as alternative or auxiliary carriers for microbial formulations. These materials generally exhibit good biocompatibility, film-forming capacity, and tunable degradation properties, which may help improve strain encapsulation efficiency, shelf life, and environmental release stability (Saberi Riseh et al., 2021; Hamed et al., 2024; Ali et al., 2025). However, compared with porous nano-Si, polymer carriers themselves usually do not directly provide plant-available Si. Therefore, they may be more suitable as protective coating materials or as components of composite delivery systems constructed with Si-based materials.

When developing nano-Si-based microbial inoculants, systematic ecological risk and food safety assessments should be conducted in parallel. Although nano-Si carriers and nano-enabled bioformulations have great potential to improve Si bioavailability and microbial delivery efficiency, their long-term ecological risks and food safety concerns must be carefully considered during formulation development. After repeated application, nanoparticles may affect indigenous soil microbial communities, microbial diversity, and core microbial functions involved in nutrient cycling, organic matter decomposition, and rhizosphere homeostasis, depending on their physicochemical properties, application rate, soil characteristics, and exposure duration (Dinesh et al., 2012; Simonin and Richaume, 2015; Zhi et al., 2021). In addition, nanoparticles may undergo a series of transformation processes in the soil matrix, including aggregation, dissolution, surface aging, and adsorption, and may interact with clay minerals, organic matter, agrochemicals, and metal ions. These processes can further alter their mobility, bioavailability, environmental persistence, and ecological effects (McKee and Filser, 2016; Ren et al., 2024).

Future development of Si–microorganism composite inoculants should integrate carrier optimization with long-term field trials, standardized ecotoxicological assessment, soil microbial community analysis, plant tissue residue detection, and food safety testing. Such integrated evaluation is necessary to establish environmentally safe application thresholds and relevant regulatory standards. Overall, the development of structurally optimized microbial carriers will become an important direction for nano-Si–microorganism composite systems.

Regarding release strategies, most current silicon-based microbial formulations rely on slow-release mechanisms. From a materials engineering perspective, however, future efforts should focus on developing “smart-release” systems responsive to environmental cues. For instance, by integrating nanosilicon with stimuli-responsive biopolymers, it may be possible to design composite materials sensitive to changes in soil pH, moisture, or redox conditions, thereby enabling on-demand release of silicon nutrients and functional microorganisms (Lee et al., 2022; Thirumurugan et al., 2024). In principle, such systems could adjust release rates in response to plant stress status or rhizosphere dynamics, ensuring the timely availability of nutrients and microbial functions during critical growth stages.

In addition, the industrial production and storage stability of microbial formulations are crucial for large-scale application. Future research may further explore drying and encapsulation techniques in combination with nanocarriers, such as spray drying or freeze-drying, to produce stable granular or powdered products while maintaining microbial viability (Kawakita et al., 2021; Arbaugh et al., 2022).These formulation strategies are particularly important for improving shelf life, reducing performance variability, and enhancing the practical applicability of silicon–microbe composite products under diverse soil and climatic conditions.

Nevertheless, the development of nanosilicon-based microbial formulations should be accompanied by systematic ecological and food safety assessments.Although nanosilicon carriers and nano-enabled bioformulations exhibit considerable potential for improving silicon bioavailability and microbial delivery efficiency, their long-term ecological and food safety risks should be carefully considered during formulation development. Depending on particle properties, dosage, soil characteristics, and exposure duration, repeated nanoparticle application may affect native soil microbial communities, microbial diversity, and key microbial functions involved in nutrient cycling, organic matter decomposition, and rhizosphere homeostasis (Dinesh et al., 2012; Simonin and Richaume, 2015; Zhi et al., 2021). Moreover, nanoparticles may undergo aggregation, dissolution, surface aging, sorption, or other transformation processes in soil matrices, and may interact with clay minerals, organic matter, agrochemicals, and metal ions, thereby influencing their mobility, bioavailability, persistence, and ecological effects (McKee and Filser, 2016; Ren et al., 2024).

Food safety is another important consideration for nanoparticle-assisted silicon–microbe technologies. Compared with conventional silicate fertilizers, nanoscale silicon materials may exhibit distinct mobility, surface reactivity, and plant uptake behavior. Previous studies on engineered nanomaterials have shown that nanoparticles can be taken up by plants and translocated through plant tissues, although the extent of accumulation, transformation, and toxicological relevance strongly depends on nanoparticle type, plant species, exposure route, and environmental conditions (Hernandez-Viezcas et al., 2013; Le et al., 2014; Su et al., 2019; Pan et al., 2024). Therefore, whether nanosilicon particles or their transformed products can accumulate in edible tissues, alter contaminant mobility in the soil–plant system, or contribute to long-term dietary exposure remains to be systematically evaluated. Future development of silicon–microbe composite formulations should therefore integrate carrier optimization with long-term field evaluation, standardized ecotoxicological assessment, soil microbial community profiling, plant tissue residue analysis, and food safety testing to establish environmentally safe application thresholds and regulatory frameworks. Consequently, the design of structurally optimized microbial carriers will be a key direction in the development of nanosilicon–microbe composite systems.

Overall, future development of silicon–microbe composite formulations should integrate carrier optimization, smart-release design, industrial formulation technologies, and long-term safety assessment. As illustrated in **Figure 4D**, porous nanosilicon may serve as a core microbial carrier, while stimuli-responsive polymer coatings could enable environmentally triggered release. Such a strategy is expected to improve microbial survival, extend shelf life, enhance application efficiency, and support the development of more stable and scalable silicon–microbe biofertilizer systems. However, before field-scale deployment, these systems should be evaluated through long-term field trials, standardized ecotoxicological assays, soil microbial community profiling, plant tissue residue analysis, and food safety testing to establish environmentally safe application thresholds and regulatory frameworks.

## 7 Conclusions

In summary, silicon and beneficial microorganisms do not function as two independent regulatory pathways in alleviating abiotic stress in plants; rather, they constitute a multi-level synergistic system involving structural reinforcement, maintenance of ionic homeostasis, redox regulation, rhizosphere process reconfiguration, and coupled signaling networks. This review systematically synthesizes their synergistic mechanisms across physiological and biochemical, molecular regulatory, and rhizosphere ecological levels. It further highlights the potential regulatory roles of silicon in microbial recruitment, colonization, and functional maintenance, as well as the reciprocal contributions of microorganisms to silicon activation, uptake, and internal distribution.

These insights provide a more integrative and explanatory framework for understanding silicon-mediated rhizosphere restructuring, cross-scale signal communication, and synergistic stress-resistance networks, and lay a foundation for future studies employing multi-omics approaches to identify key regulatory nodes and construct testable interaction models. At the same time, this framework offers new perspectives for improving field-level stability of stress resistance, optimizing nutrient utilization, and reducing reliance on chemical inputs—particularly in agricultural systems characterized by frequent combined stresses and increasing soil heterogeneity.

Looking forward, advances in nanosilicon carriers, intelligent composite formulations, and machine learning-assisted optimization of application strategies are expected to drive the transition of silicon–microbe synergistic technologies from empirical use toward precision design. This progress will provide critical support for the development of sustainable agricultural systems that are more resilient, resource-efficient, and environmentally friendly.
